# Global socioeconomic inequalities in vaccination coverage, supply, and confidence

**DOI:** 10.1038/s41541-025-01143-8

**Published:** 2025-05-09

**Authors:** Qiang Wang, Kathy Leung, Mark Jit, Joseph T. Wu, Leesa Lin

**Affiliations:** 1 Laboratory of Data Discovery for Health (D24H), Hong Kong Science Park, Hong Kong Special Administrative Region, Hong Kong, China; 2 Department of Infectious Disease Epidemiology, London School of Hygiene & Tropical Medicine, London, UK; 3 WHO Collaborating Centre for Infectious Disease Epidemiology and Control, School of Public Health, Li Ka Shing Faculty of Medicine, The University of Hong Kong, Hong Kong Special Administrative Region, Hong Kong, China; 4 The Hong Kong Jockey Club Global Health Institute, Hong Kong Special Administrative Region, Hong Kong, China; 5 The University of Hong Kong—Shenzhen Hospital, Shenzhen, China

**Keywords:** Public health, Infectious diseases

## Abstract

Sustainable Development Goal (SDG) adopted in 2015 aim to reduce inequalities and achieve universal health coverage, including access to essential vaccines for all. Using data from WHO, the Vaccine Confidence Project™, World Bank, and UNDP, we analyzed between-country inequalities in coverage of four vaccines (DTP1, DTP3, MCV1, and POL3), vaccine stock-outs, and vaccine confidence. Economic- and education-related inequalities in coverage (measured by the concentration index) declined from 2015 to 2019, increased in 2020, peaked in 2021, and have declined again since 2022. Inequalities increased continuously in the Region of the Americas. Over 2015–2022, 94 countries/territories reported at least one national level DTP-containing vaccine stock-out. Countries/territories with higher income or education attainment showed lower vaccine confidence. Our study underscores the decrease of inequalities in vaccination coverage following the SDG adoption in most regions, and emphasizes the need to address vaccine stock-outs and strength the vaccine confidence.

## Introduction

Vaccination is widely recognized as one of the most effective and cost-effective public health interventions, playing a crucial role in preventing infectious diseases worldwide. Since 1980, there has been a notable increase in the global coverage of routine vaccines^1^. In 2023, ~84% of infants worldwide received three doses of the diphtheria-tetanus-pertussis (DTP3) vaccine^1^. However, this overall figure masks substantial disparities in vaccination coverage between countries of different income levels. Low-income countries (LICs) continue to lag behind in vaccination coverage, highlighting persistent between-country inequalities^2^.

Sustainable Development Goal (SDG) 3.8, adopted by the United Nations (UN) in 2015, aims to achieve universal health coverage, including access to essential vaccines for all by 2030^3^. Reducing inequalities and ensuring no one is left behind are integral to SDG 10, “Reduced Inequalities.” Understanding the changes in socioeconomic-related between-country inequalities in vaccination coverage since the adoption of SDG, both globally and regionally, is crucial for developing remedial interventions. Additionally, the COVID-19 pandemic severely disrupted routine immunization services worldwide^4^. Consequently, between-country inequalities in vaccination coverage for 11 childhood vaccines increased across 195 countries/territories between 2020 and 2021, compared to 2019^5^. Global vaccination coverage began to recover in 2022, but changes in between-country inequalities remain unclear.

Vaccination coverage is influenced by various factors, which can be categorized into physical factors (related to vaccine cost, supply and logistics, and convenience of access) and attitudinal factors (related to perceptions of vaccination)^6^. Immunization expenditure has been found to be a particularly influential factor^7^. Significant differences exist between country income groups in reported spending levels on immunization^8^. Wealthier countries are likely to spend more on immunization per capita, with governments increasingly covering a larger share of the total immunization expenditure. However, immunization expenditure alone does not adequately explain the inequalities in vaccination coverage^9^. For instance, countries with similar levels of immunization spending per surviving infant can exhibit notable differences in the proportion of unvaccinated or undervaccinated children^9^. Additional associated factors need to be examined further.

Besides government expenditure, vaccine uptake is influenced by supply resilience and vaccine confidence. Strong supply chains are a prerequisite to vaccination. Vaccine stock-outs or shortages occur when there are issues with supply and logistics. Between 2011 and 2015, an average of 58 countries annually reported at least one national-level stockout event for one or more vaccine^10^. Studies have shown that vaccine stockouts varied greatly across countries^10^. Previous researches on vaccine stock-outs after the adoption of the SDGs has primarily focused on specific regions^11^ or high-income settings^12^, lacking a comprehensive global assessment. Moreover, most studies were conducted before the COVID-19 pandemic, resulting in a gap in understanding how the pandemic has influenced vaccine stock-outs on a global scale.

Additionally, disparities in individuals’ perceptions of vaccination (attitudinal factors) existed between countries, with European countries reporting lower levels of vaccine confidence comparing to African countries^13^. Limited research has examined socioeconomic-related between-country inequalities in vaccine supply and vaccine confidence. Investigating global within- and between-country distribution of these factors may provide valuable insights into the drivers of inequalities in vaccination coverage.

The vision of the Immunization Agenda 2030 (IA2030) is to create “a world where everyone, everywhere, at every age, fully benefits from vaccines for good health and well-being^14^.” Equity serves as the core of IA2030. Our study aimed to assess the changes to the between-country inequalities in coverage of four essential vaccines, as recommended by World Health Organization (WHO), at the global and regional levels after the adoption of the SDGs in 2015. Four vaccines including 1st and 3rd doses of DTP (DTP1 and DTP3), 1st dose of measles containing vaccine (MCV1), and 3rd dose of polio vaccine (POL3). We further explored the factors driving inequalities in vaccination coverage, focusing on socioeconomic-related inequalities in vaccine supply and vaccine confidence.

## Results

### Vaccination coverage analysis

**Fig. 1:**
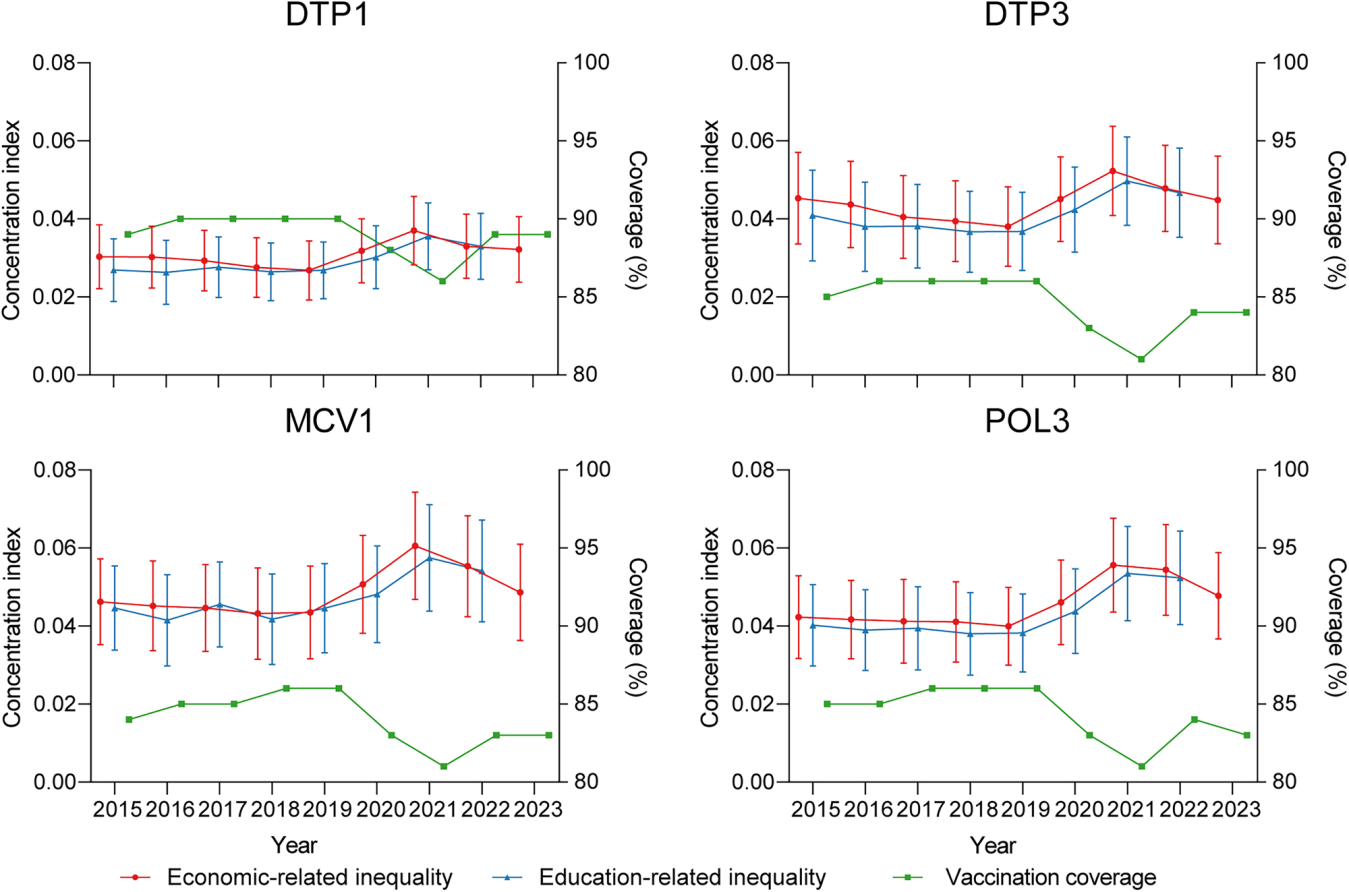
Global economic- and education-related between-country inequalities in vaccination coverage. Red lines show the economic-related CI with error bar representing 95% confidential interval. Blue lines show the education-related CI with error bar representing 95% confidential interval. Green lines indicate the vaccination coverage across all WHO member countries/territories. Positive CI values denote higher vaccination coverage among countries with more wealth or higher education. CI concentration index.

Globally, coverage for four vaccines showed a slight upward trend from 2015 to 2019 (DTP1: from 89% to 90%; DTP3 and POL3: from 85% to 86%; MCV1: from 84% to 86%) (Fig. 1 and Supplementary Fig. 1). However, coverage declined between 2019 and 2021, reaching its lowest point since 2015 in 2021 (DTP1: from 90% to 86%; DTP3, MCV1, and POL3: from 86% to 81%). In 2022, coverage increased for all four vaccines (DTP1: from 86% to 89%; DTP3 and POL3: from 81% to 84%; MCV1: from 81% to 83%). In 2023, the coverage of DTP1, DTP3, and MCV1 remained unchanged from 2022, while POL3 coverage showed a slight decline (from 84% to 83%). For the economic-related inequality analysis, data from 189 (96.9% of the 194 WHO Member States) countries/territories were included, while for the education-related inequality analysis, data from 191 (98.5%) countries/territories were used. Global economic- and education-related between-country inequalities in vaccination coverage were observed, with a statistically significant (alpha = 0.05) positive concentration index (all *p* < 0.001). From 2015 to 2019, the economic-related inequalities between countries for the coverage of four vaccines decreased, with the largest reduction observed in DTP3 (Fig. 1 and Supplementary Table 1). Over the same period, education-related between-country inequalities declined for DTP3 and POL3 coverage, remained stable for DTP1 coverage, and fluctuated for MCV1 coverage. However, these inequalities across all four vaccines increased in 2020, peaked in 2021, and then declined in 2022. The economic-related inequalities further decreased in 2023 relative to 2022.

The African Region (AFR) had the lowest vaccination coverage, with over 30% of countries/territories reporting coverage below 75% for DTP3, MCV1, and POL3 (*n* = 47, Supplementary Fig. 2). In the Region of the Americas (AMR), there was a declining trend in vaccination coverage, with Haiti and Venezuela having notably lower DTP3 and POL3 coverage compared to other countries/territories (*n* = 35, Supplementary Fig. 3). 82% and 92% of countries/territories reported coverage for the four vaccines over 75% in the South-East Asia Region (SEAR) (*n* = 11) and European Region (EUR) respectively (*n* = 53) (Supplementary Figs. 4, 5). Somalia in the Eastern Mediterranean Region (EMR) and Papua New Guinea in the Western Pacific Region (WPR) exhibited notably lower vaccination coverage compared to other countries/territories in their respective region (Supplementary Figs. 6, 7). From 2015 to 2019, economic- and education-related inequalities between WHO regions gradually declined (Supplementary Table 2). However, inequalities increased in 2020 and 2021 before decreasing again in 2022. In 2023, a slight increase in economic-related inequalities in DTP3, MCV1, and POL3 coverage was observed compared to 2022.

**Fig. 2:**
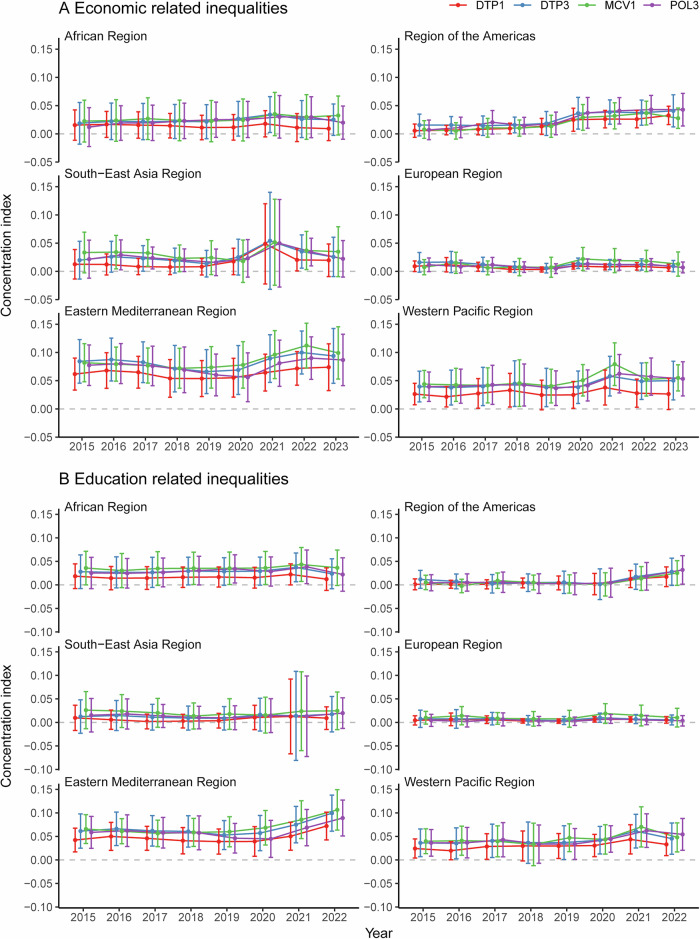
Regional economic- and education-related between-country inequalities in vaccination coverage. **A** the economic-related inequalities in vaccination coverage between countries. **B** the education-related inequalities in vaccination coverage between countries. Positive CI values denote higher vaccination coverage among countries with more wealth or higher education. The gray dashed line represents the statistically invalid line. The CI with 95% confidential interval crossing the gray line show the inequality was not statistically significant. CI concentration index.

EMR exhibited the highest levels of economic- and education-related inequalities, followed by the WPR, while the EUR demonstrated the lowest levels of inequalities (Fig. 2). Economic- and education-related between-country inequalities in six WHO regions did not show a similar trend between 2015 and 2023. Before 2020, economic-related inequalities in coverage of four vaccines exhibited a decreasing trend in the EUR, SEAR, EMR, and WPR, while showing an increasing trend in the AMR. The economic-related inequalities in coverage of DTP3 and POL3 exhibited an increasing trend in the AFR. In 2020, compared to 2019, economic-related inequalities increased in six WHO regions. In 2021, economic-related inequalities decreased in the EUR compared to 2020, but increased in other WHO regions. By 2022, economic-related inequalities had decreased compared to 2021 in AFR, SEAR, and WPR, but continued to rise significantly in the AMR and EMR. The economic-related inequalities in DTP1 and DTP3 coverage had increased in the AMR in 2023 than 2022. The education-related inequalities in coverage of four vaccines increased in the AMR and EMR since 2020. Between-country inequalities were statistically significant (*p* < 0.001) in the EMR and WPR between 2015 and 2023 (Supplementary Tables 3, 4). Sensitivity analyses revealed that the values and trends of economic-related inequalities at both global and regional levels, calculated using GDP per capita and GDP per capita in PPP, were closely aligned (Supplementary Fig. 8 and Tables 5, 6).

The coverage of four vaccines in LICs was lower than that in high-income-countries (HICs), upper-middle-income countries (UMICs), and lower-middle-income countries (LMICs). The coverage of four vaccines in LICs exhibited a downward trend from 2015 to 2021, followed by an increasing trend from 2021 to 2023 (Supplementary Fig. 9 and Table 7). From 2015 to 2021, inequalities between country-income groups gradually increased (Supplementary Table 8). Since 2022, economic-related inequalities have shown a decline. Economic-related between-country inequalities within country-income groups were not statistically significant (Supplementary Table 9). Statistically significant education-related between-country inequalities in MCV1 (concentration index: 0.032 to 0.051) and POL3 coverage (concentration index: 0.023 to 0.039) were observed in the LMICs between 2015 and 2022 (*p* < 0.001) (Supplementary Table 10). In 2015 and 2016, the concentration index (below zero) associated with education in DTP1, DTP3, and POL3 coverage was statistically significant (*p* < 0.001) in the HICs.

### Vaccine supply analysis

**Fig. 3:**
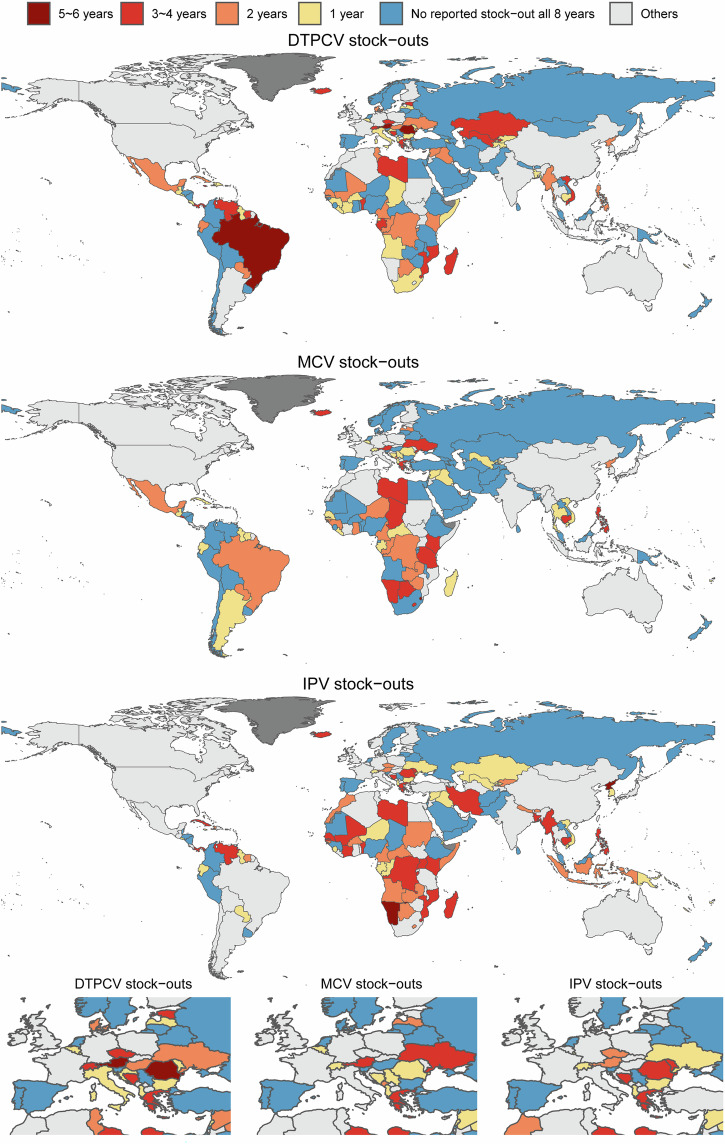
The reported frequency of national-level vaccine stock-outs from 2015 to 2022. The figure illustrates the occurrence of DTPCV, MCV, and IPV stock-outs at the national level between 2015 and 2022. Categories are defined as follows: “5–6 years” indicates that the country experienced the vaccine stock-outs for 5 or 6 of the 8 years; “3–4 years” denotes stock-outs for 3 or 4 years; “2 year” denotes stock-outs in 2 years; “1 year” denotes stock-outs in a single year. “No reported stock-out all 8 years” indicates that the country did not report any stock-outs during this period. Three horizontally aligned subplots at the bottom are included to illustrate stock-outs in Europe. Base map data from Natural Earth (public domain), rendered using R packages rnaturalearth, rnaturalearthdata, and sf.

After excluding “No Response” (NR), “No data” (ND), and missing values (retaining only “Yes” or “No” responses), 195 countries/territories reported DTP-containing vaccine (DTPCV) and MCV stock-out status for at least 1 year between 2015 and 2022, and 185 countries/territories did so for inactivated polio vaccine (IPV) (Supplementary Tables 11, 12). A higher proportion of countries/territories in LICs, LMICs, and UMICs provided DTPCV and MCV stock-outs information compared to HICs. Across all four income groups, the proportion of countries/territories reporting stock-outs status in 2020 was lower than in 2019. Over 2015–2022, 94 countries/territories reported at least one DTPCV stock-out, 76 reported at least one MCV stock-out, and 87 reported at least one IPV stock-out at the national level (Fig. 3). Among them, four countries (Austria, Brazil, Dominica, and Romania) reported DTPCV stock-outs in more than five of the 8 years; two (Dominica and Swaziland) did so for MCV, and two (North Korea and Namibia) for IPV. Additionally, 83 of 154 (providing stock-out information) countries/territories reported at least one stock-out of home-based vaccination records (HBR) for children and/or women between 2015 and 2023, with 12 reporting stock-outs for more than 5 of the 9 years (Supplementary Fig. 10).

### Vaccine confidence analysis

**Fig. 4:**
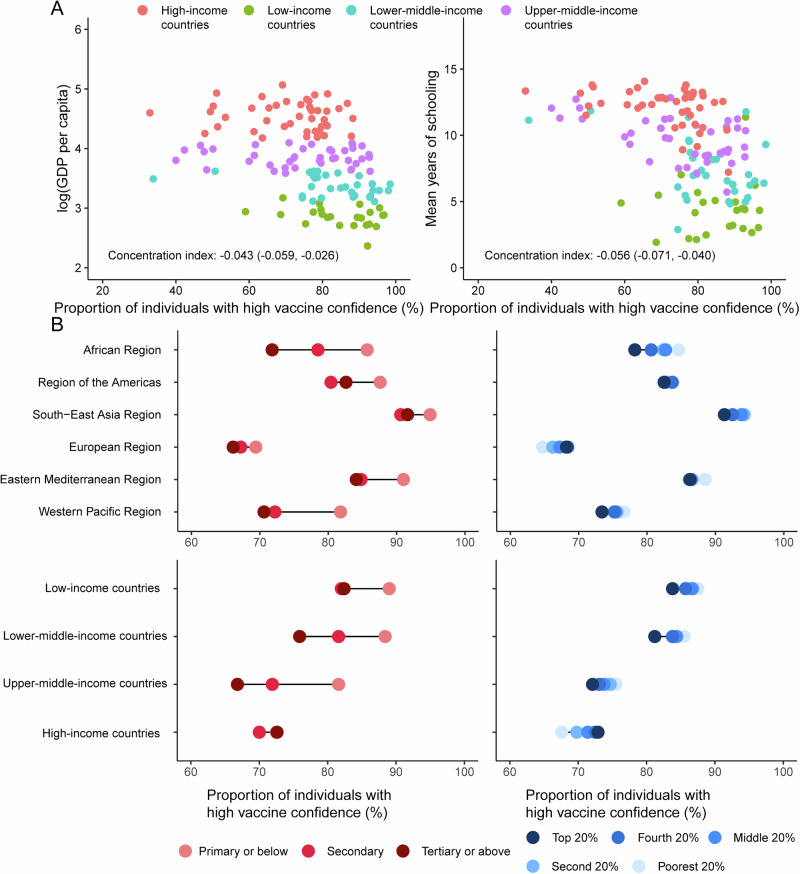
Vaccine confidence by education and income. **A** Scatter plot shows the proportion of individuals with high vaccine confidence and socioeconomic status at the national level. For presentation purposes, we use log-transformed values of GDP per capita. **B** Data are proportion of individuals with high vaccine confidence by individuals’ sociodemographic at WHO regions and country-income groups.

A total of 122,146 individuals from 141 countries were included, with the global proportion of individuals with high vaccine confidence standing at 77.00% (Supplementary Table 13). The proportion of individuals with high vaccine confidence was the highest in the SEAR and the lowest in the EUR (Supplementary Fig. 11). Additionally, disparities in proportion of individuals with high vaccine confidence were noted across country-income groups, with the highest proportion observed in LICs and the lowest in the HICs. For the economic-related inequality analysis of vaccine confidence, data from 137 countries were included, while for the education-related inequality analysis, data from 138 countries were used. Globally, the socioeconomic-related between-country inequalities in vaccine confidence were statistically significant (*p* < 0.001) (Fig. 4). Countries/territories with higher GDP per capita or mean years of schooling were more likely to show lower vaccine confidence. In the LMICs (concentration index: −0.034, 95% confidential interval [95% CI]: −0.066, −0.002, *p* = 0.039), UMICs (concentration index: −0.064, 95% CI: −0.096, −0.032, *p* < 0.001), and HICs (concentration index: −0.037, 95% CI: −0.066, −0.008, *p* = 0.014), education-related between-country inequalities were statistically significant (Supplementary Table 14). In the AFR, economic-related concentration index was −0.030 (95% CI: −0.052, −0.008, *p* = 0.009).

**Fig. 5:**
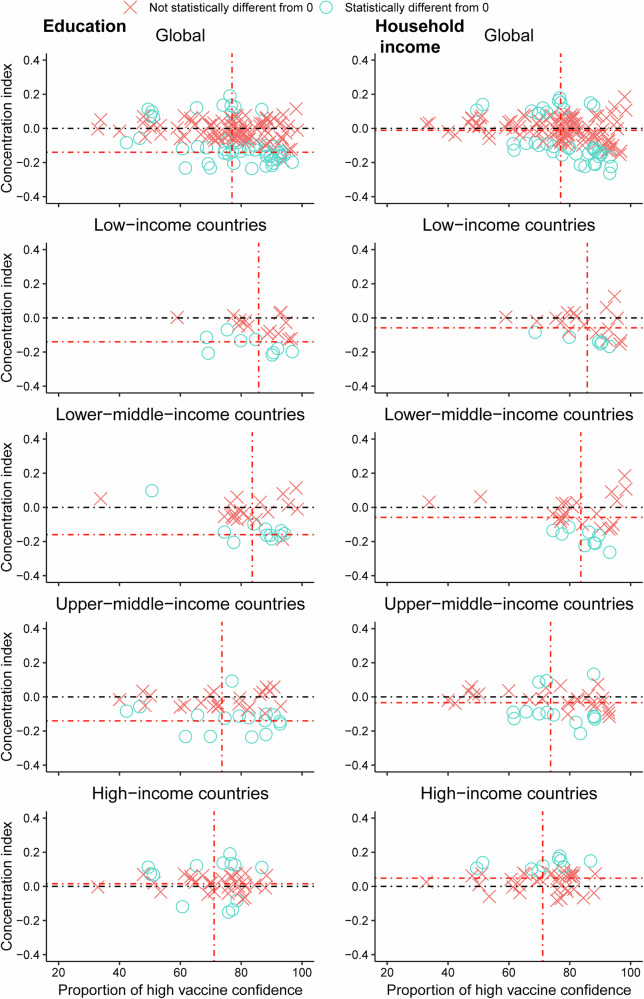
Economic- and education- related within-country inequalities in vaccine confidence. Data are within-country inequalities in vaccine confidence compared with national proportion of individuals with high vaccine confidence. Red dashed lines show the global and regional between-country inequalities and proportion of individuals with high vaccine confidence. Black dashed line represents no inequality. Positive CI values denote higher vaccine confidence among richer or more educated individuals, and negative CI values denote higher vaccine confidence among poorer or less educated individuals. A blue circle means that the CI is statistically significant and a red cross means that the CI is not statistically significant. CI concentration index.

There were 49 countries/territories exhibiting statistically significant education-related within-country concentration indices, with 38 having an index below zero and 11 above zero (Fig. 5 and Supplementary Figs. 12, 13). Income-related within-country inequalities with statistical significance were observed in 41 countries/territories, with index below zero in 28 and above zero in 13. The pro-less-educated and pro-poor within-country inequalities in vaccine confidence (higher vaccine confidence among less-educated/poorer populations) were more likely to be detected in the LICs, LMICs, and UMICs. Within the HICs, nine countries/territories reported pro-well-educated within-country inequalities, while four exhibited pro-less-educated within-country inequalities. There were ten countries/territories reporting pro-rich within-country inequalities in high vaccine confidence within the HICs.

## Discussion

Our study demonstrated that the global income- and education-related between-country inequalities in vaccination coverage showed a downward trend from 2015 to 2019. However, this progress was disrupted during the first 2 years of COVID-19 pandemic, with a decrease in coverage and an increase in inequalities, consistent with previous findings^5^. Encouragingly, there was a recovery in vaccination coverage and a reduction in these inequalities since 2022.

In the AMR, there was an observable downward trend in the coverage of four vaccines alongside an increase in economic- and education-related between-country inequalities. Notably, many countries exhibiting this declining trend were from Latin America and the Caribbean. Some countries in the region have fragile primary health-care systems characterized by inadequate staffing and an incapacity to meet the heightened demand for vaccination services^15^. Moreover, some countries, such as Venezuela, experienced a reduction in government expenditure on vaccines procurement^16^. Additionally, vaccine hesitancy has emerged as a pertinent factor associated with the decline in coverage^17^.

Maintaining adequate vaccine supply has remained a challenge^18^. Approximately 100 countries/territories worldwide reported being affected by vaccine stockouts from 2015 to 2022. These stockouts were influenced by a multitude of factors, encompassing both supply-side challenges, such as interruptions in production and a constrained supplier base, as well as demand-side dynamics, including change in vaccination program requirements^12,19^. Additionally, the delayed exchange of information between supply and demand may further exacerbate vaccine supply challenges^20^. LMICs, UMICs, and HICs were more affected by DTP and MCV stock-outs before 2020 compared to LICs. One possible explanation is that, unlike Gavi-eligible LICs, these countries (not including Gavi-supported middle income countries) need to procure vaccines independently^9,21^. Additionally, funding constraints might pose greater challenges in LMICs and UMICs than in HICs. The impact of COVID-19 on vaccine stockouts differed across income groups. LICs were more affected than LMICs and UMICs, likely due to their greater reliance on external funding and increased vulnerability to supply chain disruptions. Monitoring of vaccine stock-outs was inadequate in HICs, where the data on vaccine stock-outs were unavailable in more than 20% of countries/territories. This suggests that many HICs don not have necessarily a centralized way to track vaccine stock-outs. Within the LICs, the monitoring capability of vaccine stock-outs was reassuring, as evidenced by over 90% of countries/territories consistently reporting DTPCV and MCV stock-outs data. This may be attributed to Gavi-supported LICs regularly collecting and reporting supply chain information.

The countries characterized by lower income levels or education attainment were more likely to show higher vaccine confidence. However, within-country inequalities in vaccine confidence by socio-economic status showed contrasting trends between HICS and other three country-income groupings. Individuals with higher education levels or household income in HICs were more likely to show higher level of vaccine confidence. Conversely, higher levels of vaccine confidence were more prevalent among individuals with lower education levels and household incomes within LICs, LMICs, and UMICs. This is consistent with findings in a previous study on COVID-19 vaccine hesitancy^6^. Bergen et al. suggested that these differences stemmed from varying responses to misinformation and disinformation among individuals with different socio-demographic characteristics^22^. In HICs, individuals from socio-economic disadvantaged backgrounds may be more likely to harbor skepticism towards political and authoritative institutions^23^. This skepticism may extend to scientific establishments and experts, including healthcare services, such as vaccines, which they can access^24^. In LICs, LMICs, and UMICs, wealthy individuals often have access to a wider range of information. They may be influenced by anti-vaccine movements, especially through social media and the internet. The exposure to disinformation might contribute to lower level of vaccine confidence^25^. More efforts are needed to explore the associated factors driving vaccine confidence, which could offer insights about appropriate interventions.

The concentration indices of vaccination coverage and vaccine confidence exhibited opposite trends, suggesting that vaccine confidence may have a relatively limited role—compared to structural factors—in shaping inequalities in coverage of essential vaccines. This relationship may be influenced by contextual determinants, such as legal requirements, access to vaccination information, and cultural factors, which vary across countries. For example, in some HICs (e.g., the United States), vaccinating for children is often mandatory for school enrollment, though variable exemption rules apply^26^. Enforcement of such policies could increase and sustain vaccination coverage, thereby weakening the association between vaccination coverage and vaccine confidence. However, issue of vaccine confidence remains crucial, with declining trends observed after the COVID-19 pandemic^27^. In the long term, reduced confidence may threaten the success and effectiveness of immunization programs.

There were some limitations to our study. Firstly, the analysis is dependent on the availability and completeness of data from multiple countries/territories. The vaccination coverage estimates from the WHO and United Nations Children’s Fund (UNICEF) estimates of national immunization coverage (WUENIC) are subject to uncertainty, with potential issues such as report data quality and discrepancies in data collection methods^28^. Not all countries have survey data to validate reported administrative data. This uncertainty has not been formally quantified and hence was not accounted for in our study. The vaccine stock-outs data are based on self-reported information. Not all countries/territories report stock-outs information, which may lead to an underestimation of global vaccine stock-outs. Additionally, among countries that do report stock-outs, the duration is not specified. Moreover, the impact of national-level stock-outs on vaccine availability at the point of service remains uncertain. The Joint Reporting Process provide further details on the data reported to WHO and UNICEF^29^. Secondly, the vaccine confidence data were collected in 2018. Vaccine hesitancy is likely to have changed since then due to multiple factors, such as the COVID-19 pandemic and associated COVID-19 vaccine rollout. Thirdly, we analyzed the between-country inequalities in vaccination coverage at the global and regional levels, which limited the granularity of the analysis. Conducting more fine-grained analyses, such as within-country inequalities, could provide more valuable insights. Fourth, we employed the concentration index as it captures both the directionality and magnitude of inequalities. Moreover, unlike simple measures such as absolute differences and ratios, the concentration index is less affected by outliers^30^. However, its interpretation is less intuitive, as it does not directly reflect differences in vaccination coverage. The observed trend in the inequalities should be interpreted with caution, given that overlapping CIs of the concentration index reflect uncertainty about the statistical significance of the observed increases or decreases. Finally, we used mean years of schooling as a proxy for the educational attainment of countries/territories, which is subject to methodological limitations as an indicator of the national educational level^31^. The analysis of education-related inequality for 2023 was not conducted because the most recent mean years of schooling data is only available up to 2022.

Through quantifying the socioeconomic-related between-country inequalities in vaccination coverage, our study underscores progress in coverage of essential vaccines towards achieving the vision of equality in SDG globally. We also identified socioeconomic-related inequalities in vaccine supply and vaccine confidence. The COVID-19 pandemic affected global vaccine stock-outs, especially in LICs. The trend of within-country inequalities in vaccine confidence varied among country-income groupings. However, vaccine confidence may not be as important a driver for inequalities in coverage of essential vaccine as physical factors at this time. Further research is needed to explore additional physical factors contributing to the inequalities in vaccination coverage, providing insights necessary for realizing the vision of SDG.

## Methods

### Data sources

Vaccination coverage data were sourced from the WUENIC. This dataset provides immunization coverage by a specific vaccine, country/territory, region, and year^1^. Estimates of vaccination coverage in WUENIC were based on administrative data and household surveys that involved checking immunization records or asking a child’s caregiver, or both^32^. The Expanded Programme on Immunization was launched in 1974 and has evolved into what is now commonly known as the Essential Programme on Immunization. It currently includes 13 vaccines recommended by WHO^33^. National immunization programs adapt these recommendations to develop schedules based on local disease epidemiology and national health priorities. The pace at which countries adopt the vaccines into their national immunization programs varies, leading to differences in schedule and the number of recommended vaccines. Only DTP, polio, and MCV are universally used across all countries/territories^34^. For comparability purpose, we extracted data on vaccination coverage for the DTP1, DTP3, MCV1, and POL3 between 2015 and 2023 from WUENIC (the 2023 revision) on March 12, 2025.

We extracted the annual occurrence of national stock-out of DTPCV, MCV, and IPV, as well as the annual occurrence of stock-outs of HBR for children and/or women from the WHO database (the 2023 revision)^1^. HBR are essential for tracking individual immunizations and primary healthcare services, and a stock-out of HBR indicates a situation in which no record documents are available for distribution^35^. HBR stock-outs data were extracted for the period 2015–2023, while data on vaccine stock-outs were extracted for 2015–2022, as 2023 data for DTPCV and MCV were not yet available. We extracted records (on March 16, 2025) for the following questions:

“Was there a stock-out at the national level of DTP containing vaccines/measles containing vaccines/inactivated polio vaccine? (Yes/No/ NR/ ND)”.

“Was there a stock-out of home-based vaccination records for children and/or women (i.e., no remaining home-based records for any period of time) at the national level? (Yes/No/ NR/ ND).”

A survey conducted by the Vaccine Confidence Project^TM^ in 2018 was used to evaluate vaccine confidence levels worldwide, covering more than 140 countries/territories^13^. Approximately 1000 participants were surveyed from each country by online, telephone, and face-to-face survey methodologies. The data were weighted to match national age and sex distributions. Each individual’ s education level (primary or below vs secondary vs tertiary or above) and household income (five quantiles ranging from lowest income (poorest 20%) to highest income (top 20%)) were collected. It utilized three questions with a five-point Likert scale to measure vaccine confidence: “I think vaccines are important for children to have,” “I think vaccines are safe,” and “I think vaccines are effective.” The responses were binary coded, with a “strongly agree” and “agree” response coded as “1” and all other responses coded as “0.” Participants who answered “1” to all three questions were classified as having “high vaccine confidence,” while those who did not were classified as having “lower vaccine confidence.” Observations with at least one missing variable were removed.

Income and education are two main aspects indicating socioeconomic status^36^. Annual GDP per capita between 2015 and 2023 (the 2025 revision), derived from the World Bank, served as the income indicator for countries/territories. Considering most of LMICs need to purchase vaccines, we also extracted GDP per capital in PPP (the 2025 revision) from the World Bank. This index adjusts for differences in the cost of living and inflation rates, allowing for more accurate between-country comparisons of economic well-being. Mean years of schooling (average number of years of education received by individuals aged 25 and older) between 2015 and 2022, was sourced from UN Development Programme (the 2024 revision) and used as the proxy for education level in the countries/territories. These data were extracted on March 12, 2025.

### Analysis

Vaccination coverage, frequency of vaccine stock-outs, and vaccine confidence were reported and analyzed at the global, regional, and national level. The regions were delineated based on six WHO regions (AFR, AMR, SEAR, EUR, EMR, and WPR), as well as four income categories (LICs, LMICs, UMICs, and HICs). Certain countries/territories that were not assigned to a specific income group were categorized as “unclassified countries” and analyzed.

The concentration index was used to measure socioeconomic-related inequalities in health outcomes^37,38^. This index is derived from a concentration curve, which plots the cumulative percentage of individuals ranked by socioeconomic status against the cumulative percentage of health outcomes. The equations of concentration index and examples of concentration curve were shown in the Supplementary Note 1 and Fig. 14. The index ranges from −1 to +1, with positive values indicating health outcome is concentrated among higher socioeconomic subgroups and negative values indicating the opposite. Higher absolute values denote greater inequality and concentration index of 0 indicates perfect equality. The economic- and education-related within-country inequalities in vaccine confidence and coverage were estimated. A concentration index greater than 0 indicates that higher vaccine confidence or coverage is more concentrated among richer or well-educated countries or populations (pro-rich/pro-well-educated), while an index below 0 suggests it is more prevalent among poorer or less-educated groups (pro-poor/pro-less-educated). When employing binary variables as the measures of health outcomes, we used the Wagstaff method to adjust the concentration index, applying sampling weights in the index calculation^39^. The concentration index and its 95% CI were calculated using Stata 14.0. Countries/territories were included in the inequality analysis only if they had available vaccination coverage data along with either economic or education data.

## Supplementary information

Supplementary information

## Data Availability

Data used in this study are publicly available. Vaccination coverage and vaccine stock-outs were available here: https://immunizationdata.who.int/. Data related to vaccine confidence were derived from the Vaccine Confidence Project^TM^: https://www.vaccineconfidence.org/vci/data-and-methodology/. National incoming data was available here: https://www.worldbank.org/en/home. Data related to mean years of schooling were available here: https://www.undp.org/.

## Code availability

The code to reproduce the results and plots of this study have been deposited in the repository under https://github.com/QiangWanglxb/Vaccine_inequality_analysis.
