# Supplementary material for: Global socioeconomic inequalities in vaccination coverage, supply, and confidence
Source: NPJ Vaccines. 2025 May 9;10:91. doi: 10.1038/s41541-025-01143-8 (PMC12064651; doi:10.1038/s41541-025-01143-8)
Supplement: Supplementary file 1 — Supplementary information [file 41541_2025_1143_MOESM1_ESM.pdf]

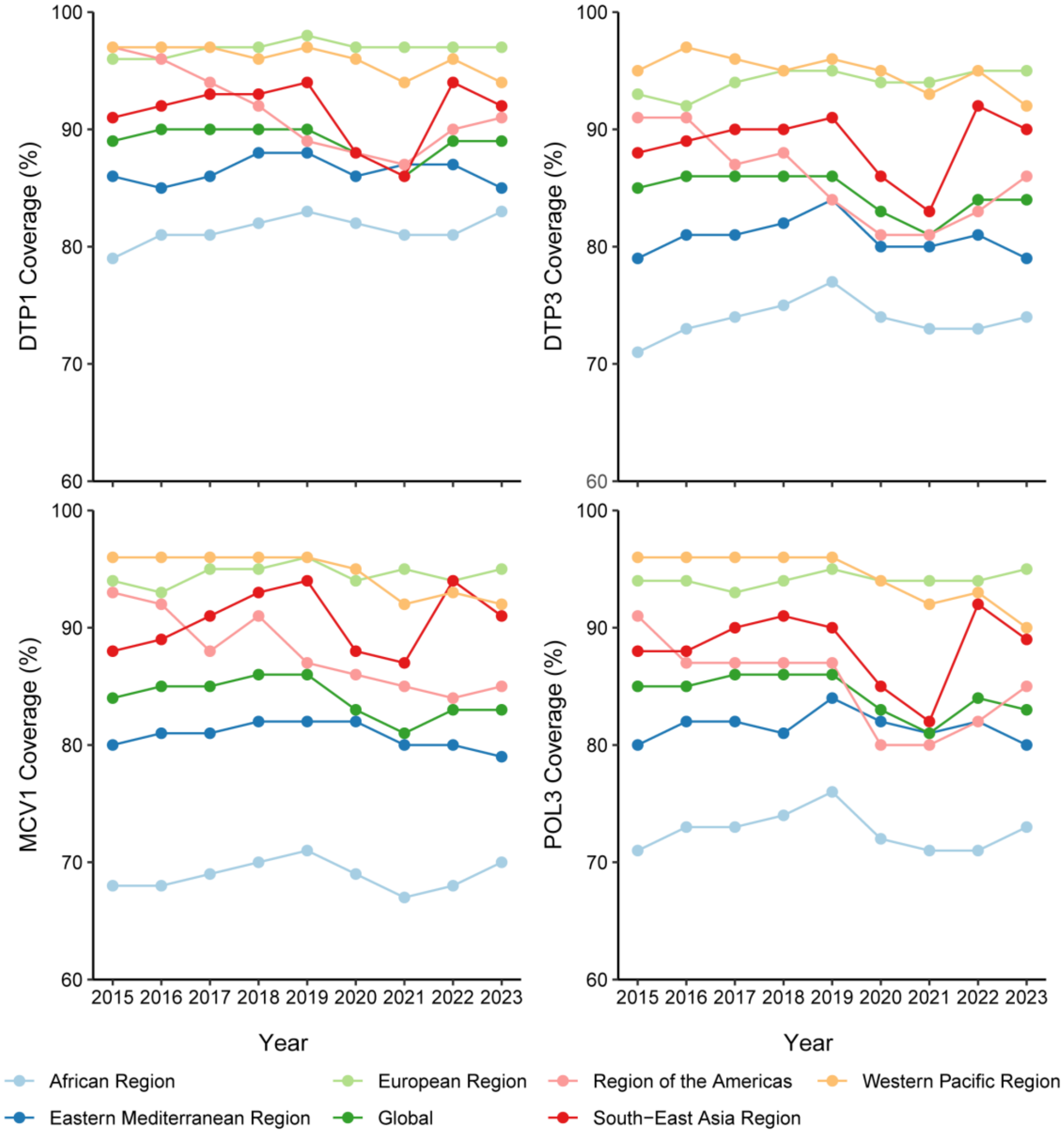

Supplementary Figure 1. Vaccination coverage by WHO region

Supplementary Table 1. Equalities between WHO regions in vaccination coverage

Type	Year	DTP1			DTP3			MCV1			POL3		
		**n**	**concentration index**	***p* value**	**n**	**concentration index**	***p* value**	**n**	**concentration index**	***p* value**	**n**	**concentration index**	***p* value**
Economic-related	2015	189	0.030 (0.022, 0.039)	< 0.001	189	0.045 (0.034, 0.057)	< 0.001	189	0.046 (0.035, 0.057)	< 0.001	189	0.042 (0.032, 0.053)	< 0.001
	2016	188	0.030 (0.022, 0.038)	< 0.001	188	0.044 (0.033, 0.055)	< 0.001	188	0.045 (0.034, 0.057)	< 0.001	188	0.042 (0.032, 0.052)	< 0.001
	2017	188	0.029 (0.022, 0.037)	< 0.001	188	0.040 (0.030, 0.051)	< 0.001	188	0.045 (0.034, 0.056)	< 0.001	188	0.041 (0.031, 0.052)	< 0.001
	2018	188	0.028 (0.020, 0.035)	< 0.001	188	0.039 (0.029, 0.050)	< 0.001	188	0.043 (0.031, 0.055)	< 0.001	188	0.041 (0.031, 0.051)	< 0.001
	2019	188	0.027 (0.019, 0.034)	< 0.001	188	0.038 (0.028, 0.048)	< 0.001	188	0.044 (0.032, 0.055)	< 0.001	188	0.040 (0.030, 0.050)	< 0.001
	2020	188	0.032 (0.024, 0.040)	< 0.001	188	0.045 (0.034, 0.056)	< 0.001	188	0.051 (0.038, 0.063)	< 0.001	188	0.046 (0.035, 0.057)	< 0.001
	2021	187	0.037 (0.028, 0.046)	< 0.001	187	0.052 (0.041, 0.064)	< 0.001	187	0.061 (0.047, 0.074)	< 0.001	187	0.056 (0.044, 0.068)	< 0.001
	2022	187	0.033 (0.025, 0.041)	< 0.001	187	0.048 (0.037, 0.059)	< 0.001	187	0.055 (0.042, 0.068)	< 0.001	187	0.054 (0.043, 0.066)	< 0.001
	2023	182	0.032 (0.024, 0.041)	< 0.001	182	0.045 (0.034, 0.056)	< 0.001	182	0.049 (0.036, 0.061)	< 0.001	182	0.048 (0.037, 0.059)	< 0.001
Education-related	2015	188	0.027 (0.019, 0.035)	< 0.001	188	0.041 (0.029, 0.053)	< 0.001	188	0.045 (0.034, 0.055)	< 0.001	188	0.040 (0.030, 0.051)	< 0.001
	2016	188	0.026 (0.018, 0.035)	< 0.001	188	0.038 (0.027, 0.049)	< 0.001	188	0.041 (0.030, 0.053)	< 0.001	188	0.039 (0.029, 0.049)	< 0.001
	2017	188	0.028 (0.020, 0.035)	< 0.001	188	0.038 (0.027, 0.049)	< 0.001	188	0.046 (0.035, 0.056)	< 0.001	188	0.039 (0.029, 0.050)	< 0.001
	2018	189	0.026 (0.019, 0.034)	< 0.001	189	0.037 (0.026, 0.047)	< 0.001	189	0.042 (0.030, 0.053)	< 0.001	189	0.038 (0.027, 0.049)	< 0.001
	2019	189	0.027 (0.020, 0.034)	< 0.001	189	0.037 (0.027, 0.047)	< 0.001	189	0.045 (0.033, 0.056)	< 0.001	189	0.038 (0.028, 0.048)	< 0.001
	2020	189	0.030 (0.022, 0.038)	< 0.001	189	0.042 (0.032, 0.053)	< 0.001	189	0.048 (0.036, 0.061)	< 0.001	189	0.044 (0.033, 0.055)	< 0.001
	2021	189	0.036 (0.027, 0.044)	< 0.001	189	0.050 (0.038, 0.061)	< 0.001	189	0.057 (0.044, 0.071)	< 0.001	189	0.053 (0.041, 0.066)	< 0.001
	2022	191	0.033 (0.024, 0.041)	< 0.001	191	0.047 (0.035, 0.058)	< 0.001	191	0.054 (0.041, 0.067)	< 0.001	191	0.052 (0.040, 0.064)	< 0.001

n represents the number of countries/territories included in the analysis.

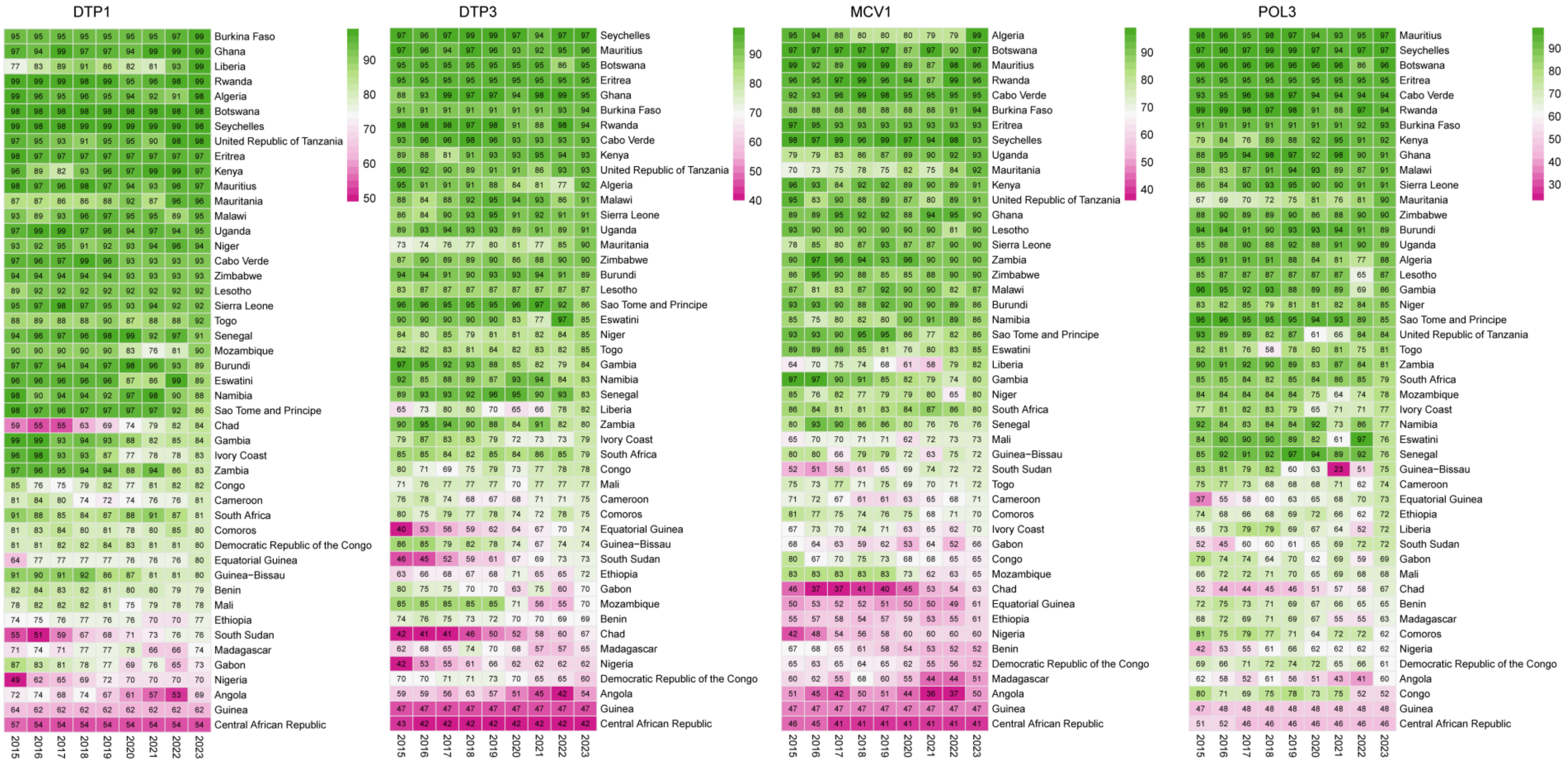

Supplementary Figure 2. Vaccination coverage in African Region

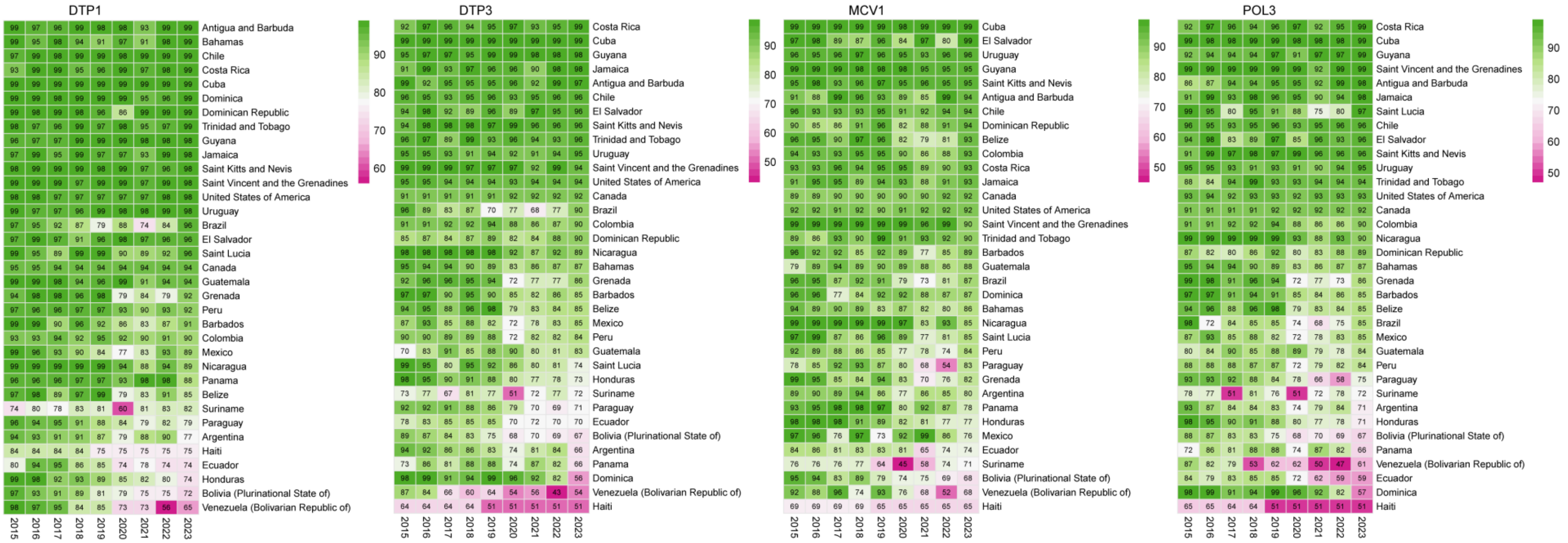

Supplementary Figure 3. Vaccination coverage in Region of the Americas

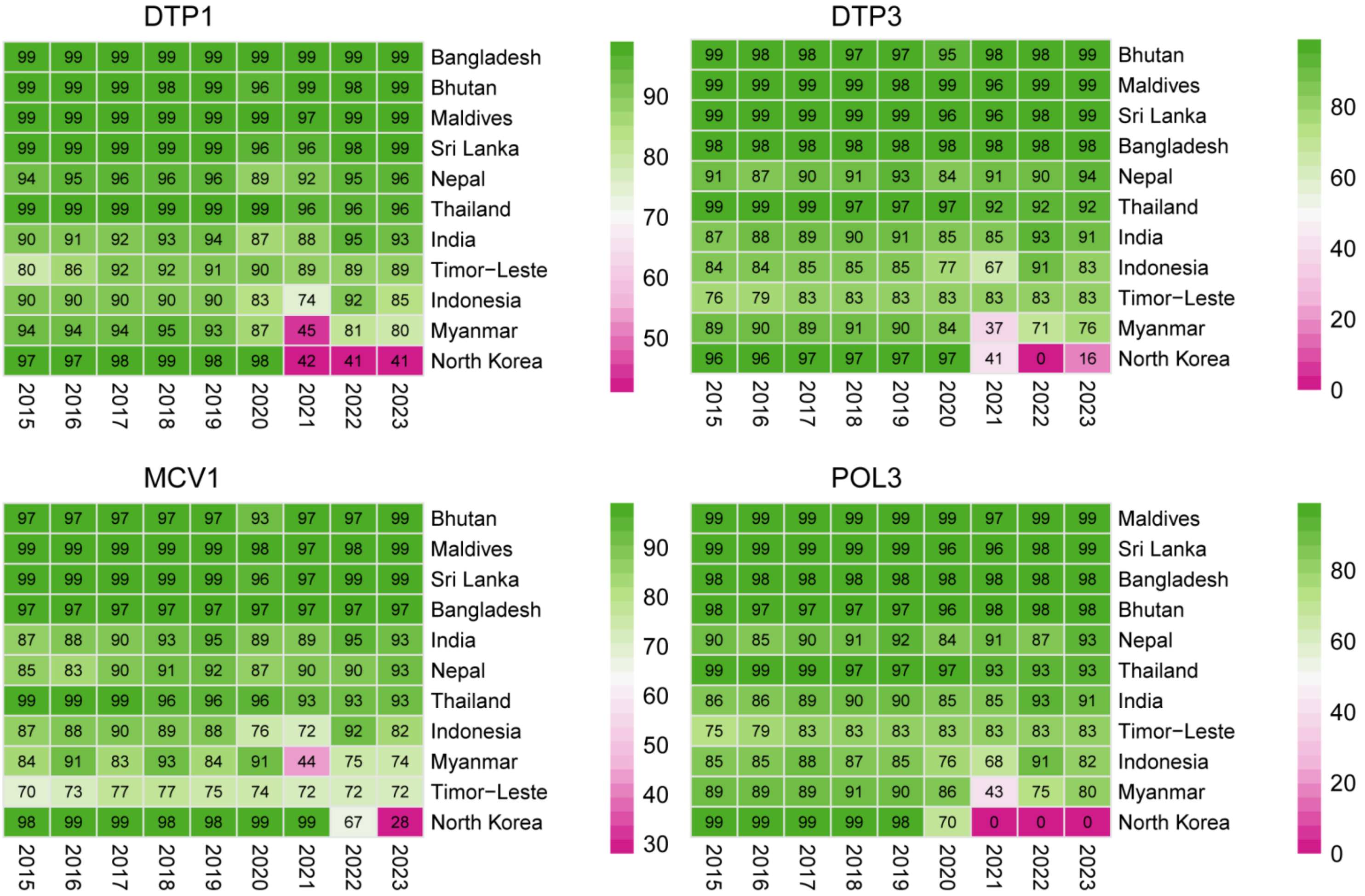

Supplementary Figure 4. Vaccination coverage in South-East Asia Region

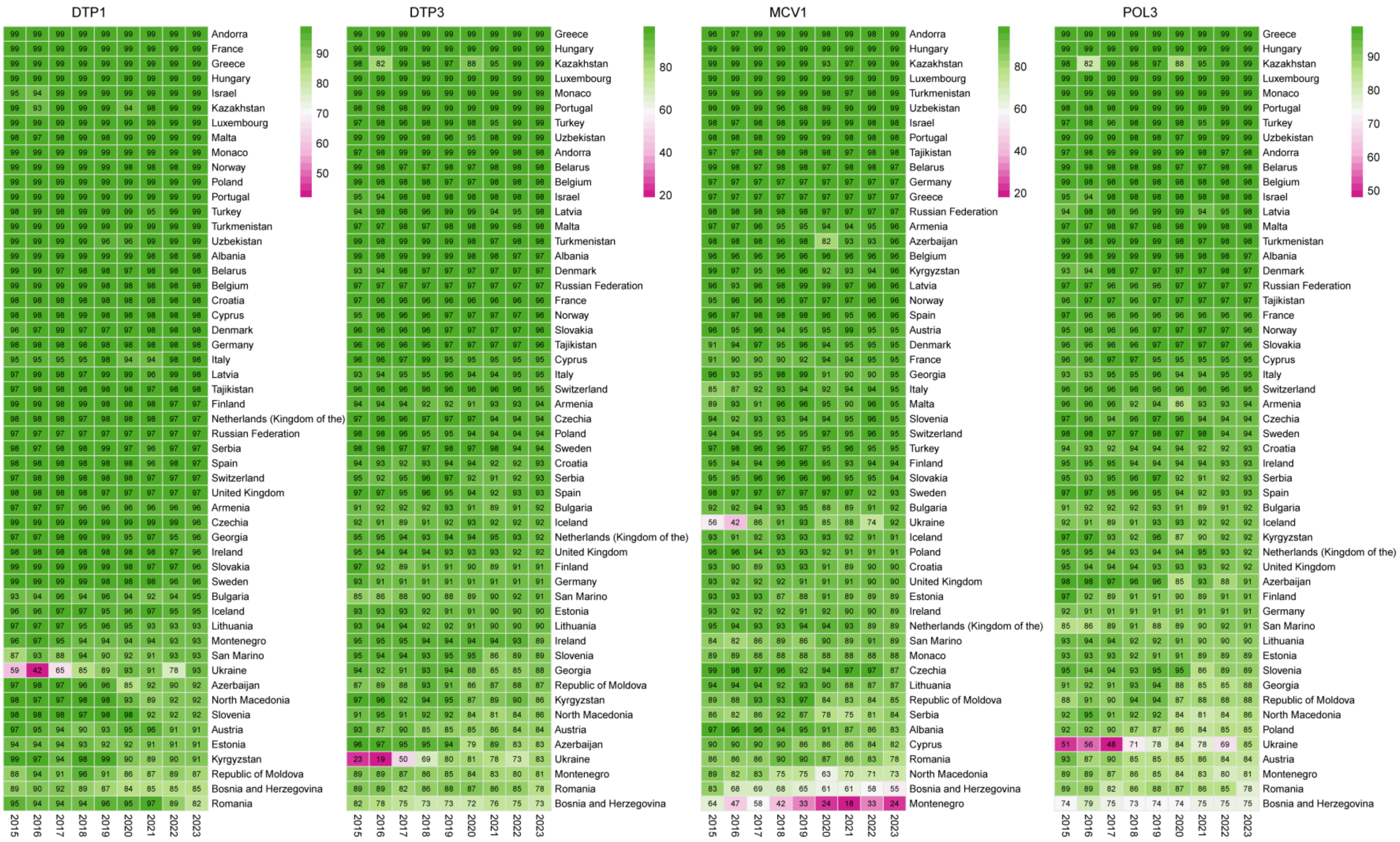

Supplementary Figure 5. Vaccination coverage in European Region

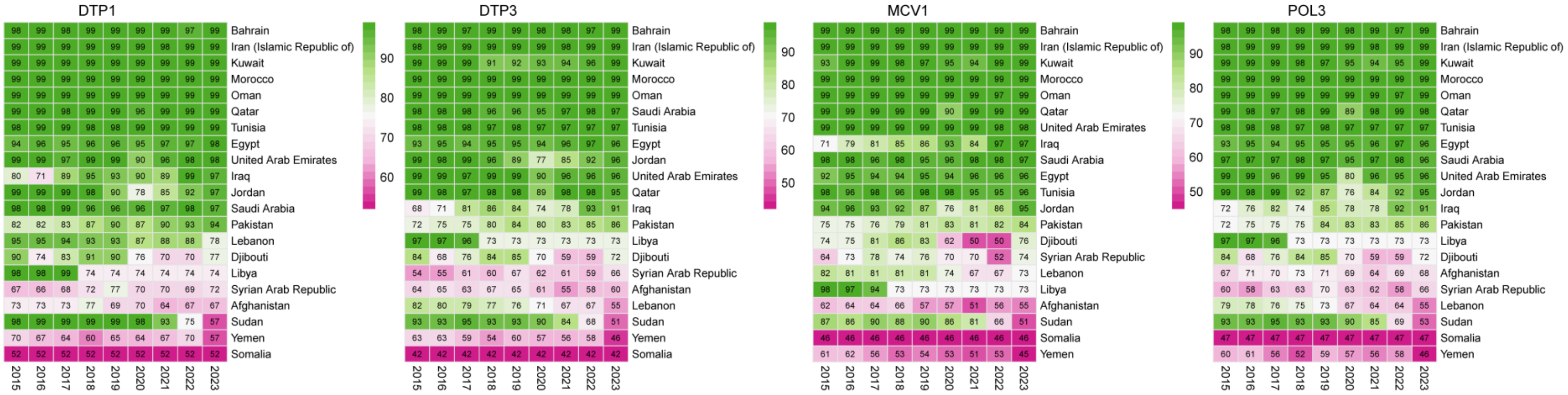

Supplementary Figure 6. Vaccination coverage in Eastern Mediterranean Region

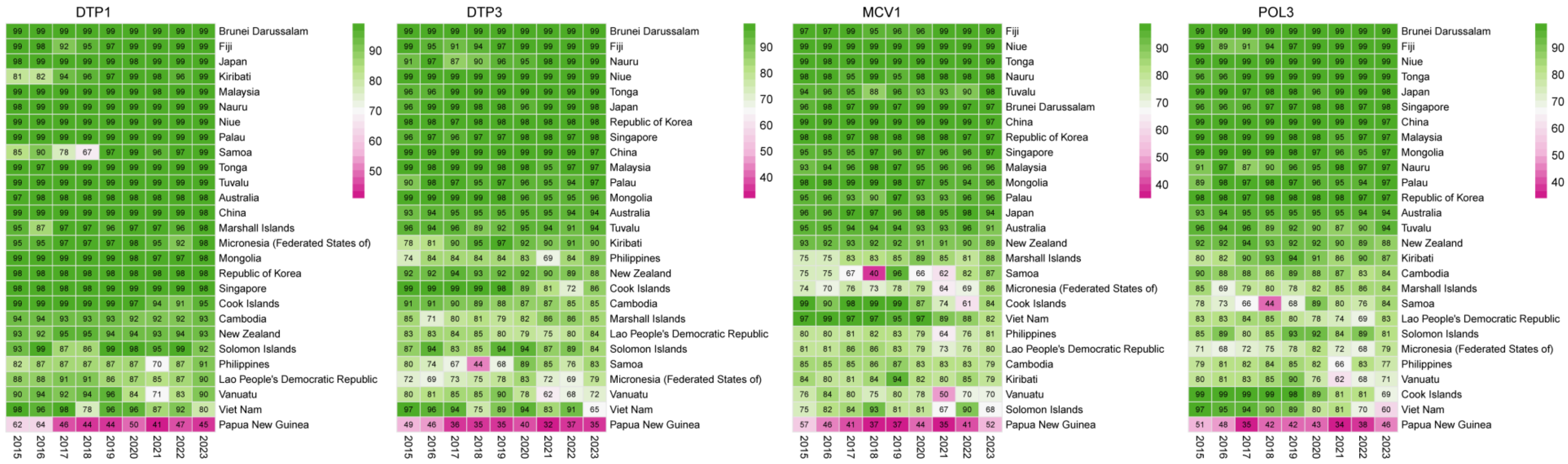

Supplementary Figure 7. Vaccination coverage in Western Pacific Region

Supplementary Table 2. Equalities between WHO regions in vaccination coverage

Type	Year	DTP1 concentration index	DTP1 *p* value	DTP3 concentration index	DTP3 *p* value	MCV1 concentration index	MCV1 *p* value	POL3 concentration index	POL3 *p* value
Economic-related	2015	0.035 (0.008, 0.062)	0.038	0.044 (0.002, 0.085)	0.070	0.052 (0.006, 0.097)	0.055	0.045 (0.003, 0.086)	0.063
	2016	0.030 (0.003, 0.057)	0.060	0.037 (-0.005, 0.080)	0.106	0.048 (0.000, 0.095)	0.076	0.037 (-0.003, 0.077)	0.093
	2017	0.029 (0.001, 0.056)	0.069	0.034 (-0.009, 0.076)	0.135	0.044 (-0.007, 0.094)	0.113	0.034 (-0.010, 0.077)	0.142
	2018	0.024 (0.000, 0.048)	0.075	0.034 (-0.003, 0.071)	0.098	0.042 (-0.008, 0.092)	0.117	0.033 (-0.012, 0.077)	0.156
	2019	0.021 (-0.009, 0.051)	0.177	0.026 (-0.016, 0.068)	0.224	0.037 (-0.016, 0.091)	0.173	0.031 (-0.006, 0.068)	0.120
	2020	0.026 (0.001, 0.052)	0.070	0.033 (-0.013, 0.078)	0.167	0.043 (-0.003, 0.089)	0.092	0.035 (-0.011, 0.081)	0.145
	2021	0.028 (0.006, 0.051)	0.043	0.037 (-0.003, 0.077)	0.093	0.048 (0.003, 0.093)	0.067	0.040 (-0.001, 0.081)	0.082
	2022	0.024 (-0.007, 0.054)	0.143	0.031 (-0.020, 0.083)	0.228	0.037 (-0.024, 0.097)	0.226	0.031 (-0.022, 0.084)	0.238
	2023	0.023 (0.000, 0.047)	0.080	0.034 (-0.007, 0.075)	0.120	0.039 (-0.011, 0.089)	0.138	0.035 (-0.003, 0.073)	0.097
Education-related	2015	0.035 (0.008, 0.062)	0.038	0.046 (0.008, 0.084)	0.045	0.054 (0.011, 0.096)	0.040	0.048 (0.012, 0.084)	0.035
	2016	0.031 (0.004, 0.057)	0.051	0.041 (0.004, 0.078)	0.059	0.050 (0.007, 0.094)	0.053	0.043 (0.012, 0.074)	0.030
	2017	0.030 (0.006, 0.055)	0.043	0.040 (0.004, 0.075)	0.057	0.049 (0.005, 0.092)	0.058	0.039 (0.003, 0.075)	0.062
	2018	0.027 (0.007, 0.047)	0.035	0.038 (0.008, 0.069)	0.042	0.045 (-0.001, 0.091)	0.080	0.039 (0.001, 0.076)	0.072
	2019	0.026 (0.001, 0.050)	0.067	0.033 (-0.001, 0.067)	0.081	0.043 (-0.004, 0.090)	0.096	0.037 (0.008, 0.065)	0.040
	2020	0.031 (0.016, 0.047)	0.009	0.042 (0.008, 0.076)	0.043	0.049 (0.012, 0.085)	0.036	0.044 (0.011, 0.077)	0.034
	2021	0.033 (0.021, 0.044)	0.003	0.045 (0.019, 0.071)	0.015	0.053 (0.015, 0.090)	0.030	0.048 (0.022, 0.074)	0.012
	2022	0.027 (0.001, 0.053)	0.068	0.039 (-0.005, 0.083)	0.104	0.043 (-0.012, 0.098)	0.142	0.038 (-0.008, 0.084)	0.121

Supplementary Table 3. Income-related between-country equalities in vaccination coverage by WHO region using GDP per capital

Vaccine	Year	African Region		Region of the Americas		South-East Asia Region		European Region		Eastern Mediterranean Region		Western Pacific Region	
		n	*p* value	n	*p* value	n	*p* value	n	*p* value	n	*p* value	n	*p* value
DTP1	2015	46	0.253	34	0.344	10	0.323	53	0.061	21	<0.001	25	0.009
	2016	45	0.152	34	0.124	10	0.190	53	0.068	21	<0.001	25	0.023
	2017	45	0.190	34	0.106	10	0.236	53	0.045	21	<0.001	25	0.044
	2018	45	0.190	34	0.049	10	0.248	53	0.125	21	0.003	25	0.033
	2019	45	0.306	34	0.068	10	0.254	53	0.150	21	0.003	25	0.068
	2020	45	0.314	34	0.019	10	0.140	53	0.001	21	0.004	25	0.044
	2021	45	0.130	33	0.002	10	0.171	53	0.004	21	0.001	25	0.020
	2022	45	0.376	33	0.002	10	0.053	53	0.009	21	<0.001	25	0.032
	2023	45	0.388	33	<0.001	9	0.177	52	0.041	19	0.002	24	0.063
DTP3	2015	46	0.317	34	0.112	10	0.232	53	0.072	21	<0.001	25	0.007
	2016	45	0.177	34	0.044	10	0.073	53	0.084	21	<0.001	25	0.020
	2017	45	0.182	34	0.100	10	0.058	53	0.055	21	<0.001	25	0.025
	2018	45	0.153	34	0.030	10	0.095	53	0.119	21	0.002	25	0.033
	2019	45	0.159	34	0.090	10	0.243	53	0.188	21	0.002	25	0.032
	2020	45	0.088	34	0.013	10	0.122	53	0.003	21	0.004	25	0.012
	2021	45	0.037	33	0.001	10	0.205	53	0.011	21	<0.001	25	0.003
	2022	45	0.116	33	0.002	10	0.031	53	0.017	21	<0.001	25	0.005
	2023	45	0.076	33	0.007	9	0.148	52	0.071	19	0.001	24	0.007
MCV1	2015	46	0.229	34	0.330	10	0.078	53	0.273	21	<0.001	25	0.001
	2016	45	0.204	34	0.474	10	0.036	53	0.112	21	<0.001	25	0.007
	2017	45	0.151	34	0.201	10	0.017	53	0.325	21	<0.001	25	0.011
	2018	45	0.193	34	0.160	10	0.067	53	0.326	21	0.001	25	0.033
	2019	45	0.221	34	0.175	10	0.115	53	0.413	21	0.001	25	0.011
	2020	45	0.193	34	0.015	10	0.327	53	0.035	21	0.001	25	0.001
	2021	45	0.073	33	0.009	10	0.177	53	0.079	21	<0.001	25	<0.001
	2022	45	0.137	33	0.001	10	0.044	53	0.057	21	<0.001	25	0.001
	2023	45	0.065	33	0.004	9	0.125	52	0.231	19	<0.001	24	<0.001
POL3	2015	46	0.491	34	0.423	10	0.200	53	0.061	21	<0.001	25	0.005
	2016	45	0.215	34	0.219	10	0.043	53	0.141	21	<0.001	25	0.012
	2017	45	0.220	34	0.056	10	0.029	53	0.080	21	<0.001	25	0.019
	2018	45	0.150	34	0.054	10	0.065	53	0.116	21	0.002	25	0.029
	2019	45	0.114	34	0.072	10	0.180	53	0.273	21	0.003	25	0.023
	2020	45	0.118	34	0.009	10	0.168	53	0.004	21	0.015	25	0.005
	2021	45	0.115	33	0.001	10	0.198	53	0.018	21	0.001	25	0.001
	2022	45	0.110	33	0.002	10	0.016	53	0.024	21	<0.001	25	0.002
	2023	45	0.181	33	0.005	9	0.172	52	0.157	19	0.001	24	0.002

n represents the number of countries/territories included in the analysis.

Supplementary Table 4. Education-related between-country equalities in vaccination coverage by WHO region

Vaccine	Year	African Region		Region of the Americas		South-East Asia Region		European Region		Eastern Mediterranean Region		Western Pacific Region	
		n	*p* value	n	*p* value	n	*p* value	n	*p* value	n	*p* value	n	*p* value
DTP1	2015	47	0.166	35	0.837	10	0.456	51	0.381	20	0.003	24	0.023
	2016	47	0.253	35	0.772	10	0.556	51	0.336	20	0.003	24	0.048
	2017	47	0.233	35	0.798	10	0.790	51	0.158	20	0.002	24	0.043
	2018	47	0.138	35	0.422	10	0.696	52	0.183	20	0.007	24	0.068
	2019	47	0.124	35	0.567	10	0.625	52	0.231	20	0.007	24	0.032
	2020	47	0.174	35	0.881	10	0.403	52	0.026	20	0.019	24	0.015
	2021	47	0.052	35	0.179	10	0.737	52	0.023	20	0.003	24	0.009
	2022	47	0.311	35	0.106	10	0.433	52	0.133	21	<0.001	25	0.009
DTP3	2015	47	0.124	35	0.225	10	0.466	51	0.410	20	0.003	24	0.019
	2016	47	0.113	35	0.347	10	0.347	51	0.459	20	0.001	24	0.039
	2017	47	0.102	35	0.812	10	0.404	51	0.238	20	0.001	24	0.028
	2018	47	0.051	35	0.617	10	0.453	52	0.345	20	0.001	24	0.101
	2019	47	0.058	35	0.643	10	0.469	52	0.350	20	0.002	24	0.047
	2020	47	0.055	35	0.932	10	0.334	52	0.046	20	0.005	24	0.008
	2021	47	0.018	35	0.198	10	0.758	52	0.145	20	0.001	24	0.002
	2022	47	0.144	35	0.060	10	0.343	52	0.326	21	<0.001	25	0.010
MCV1	2015	47	0.050	35	0.500	10	0.187	51	0.148	20	<0.001	24	0.005
	2016	47	0.100	35	0.741	10	0.172	51	0.147	20	<0.001	24	0.013
	2017	47	0.057	35	0.273	10	0.188	51	0.177	20	0.001	24	0.022
	2018	47	0.045	35	0.513	10	0.315	52	0.333	20	0.001	24	0.148
	2019	47	0.047	35	0.839	10	0.268	52	0.390	20	0.001	24	0.004
	2020	47	0.045	35	0.925	10	0.402	52	0.078	20	0.001	24	0.009
	2021	47	0.020	35	0.291	10	0.553	52	0.192	20	<0.001	24	0.003
	2022	47	0.059	35	0.060	10	0.215	52	0.314	21	<0.001	25	0.004
POL3	2015	47	0.134	35	0.593	10	0.378	51	0.497	20	0.002	24	0.015
	2016	47	0.122	35	0.580	10	0.257	51	0.730	20	0.001	24	0.029
	2017	47	0.089	35	0.600	10	0.260	51	0.358	20	0.002	24	0.024
	2018	47	0.051	35	0.789	10	0.376	52	0.548	20	0.004	24	0.105
	2019	47	0.027	35	0.843	10	0.422	52	0.584	20	0.007	24	0.045
	2020	47	0.073	35	0.763	10	0.408	52	0.119	20	0.030	24	0.004
	2021	47	0.037	35	0.221	10	0.747	52	0.313	20	0.002	24	0.002
	2022	47	0.220	35	0.041	10	0.222	52	0.533	21	<0.001	25	0.003

n represents the number of countries/territories included in the analysis.

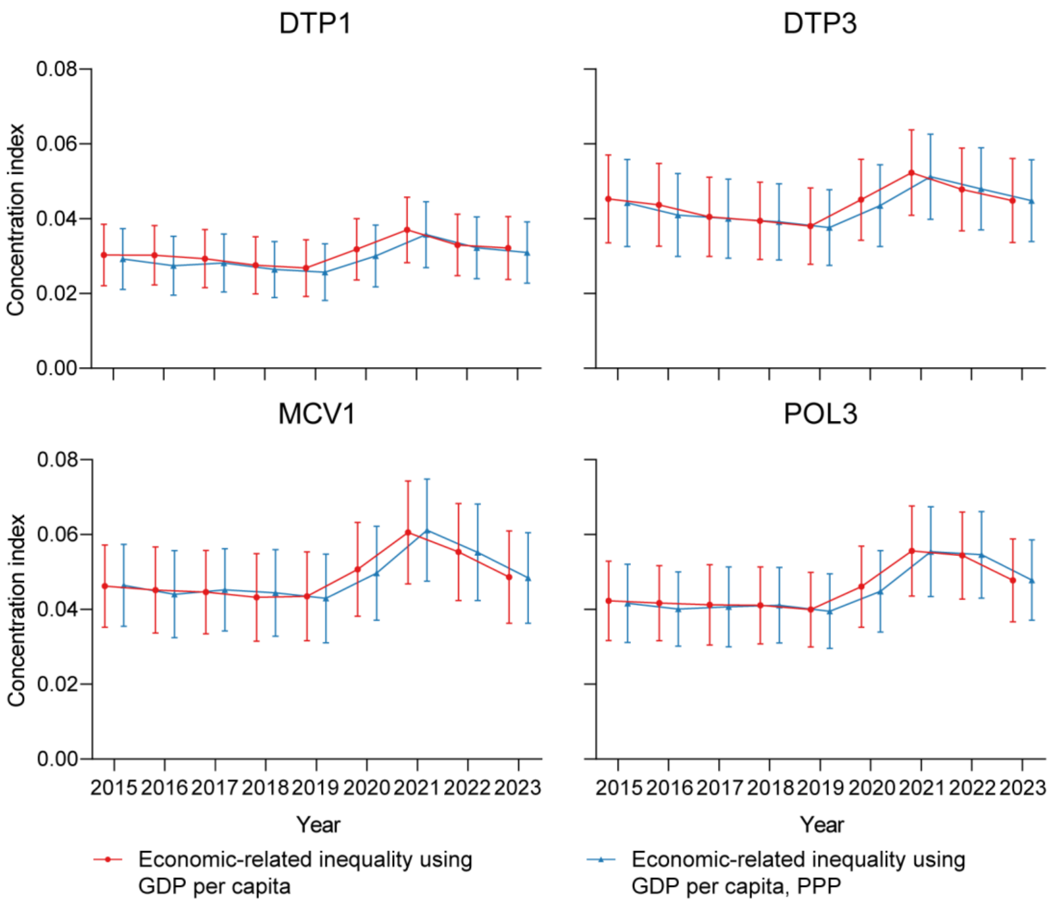

Supplementary Figure 8. Calculating concentration index using two indicators

All p values for concentration index was < 0.001.

Supplementary Table 5. Economic-related equalities between regions in vaccination coverage

Year	DTP1		DTP3		MCV1		POL3	
	**concentration index**	***p* value**	**concentration index**	***p* value**	**concentration index**	***p* value**	**concentration index**	***p* value**
WHO regions								
2015	0.024 (-0.015, 0.064)	0.221	0.029 (-0.028, 0.085)	0.300	0.036 (-0.026, 0.099)	0.245	0.029 (-0.028, 0.087)	0.293
2016	0.023 (-0.013, 0.059)	0.208	0.027 (-0.025, 0.079)	0.287	0.038 (-0.020, 0.097)	0.197	0.028 (-0.021, 0.077)	0.247
2017	0.022 (-0.013, 0.057)	0.213	0.024 (-0.027, 0.075)	0.324	0.034 (-0.026, 0.094)	0.253	0.025 (-0.026, 0.076)	0.319
2018	0.019 (-0.010, 0.049)	0.195	0.026 (-0.020, 0.071)	0.254	0.033 (-0.025, 0.091)	0.250	0.023 (-0.029, 0.075)	0.352
2019	0.015 (-0.019, 0.050)	0.349	0.018 (-0.029, 0.065)	0.418	0.029 (-0.032, 0.089)	0.328	0.023 (-0.021, 0.067)	0.279
2020	0.020 (-0.012, 0.052)	0.215	0.023 (-0.030, 0.076)	0.369	0.034 (-0.020, 0.089)	0.213	0.027 (-0.025, 0.080)	0.290
2021	0.024 (-0.005, 0.053)	0.123	0.028 (-0.020, 0.077)	0.240	0.040 (-0.015, 0.095)	0.160	0.033 (-0.016, 0.082)	0.191
2022	0.024 (-0.007, 0.054)	0.143	0.031 (-0.020, 0.083)	0.228	0.037 (-0.024, 0.097)	0.226	0.031 (-0.022, 0.084)	0.238
2023	0.023 (0.000, 0.047)	0.080	0.034 (-0.007, 0.075)	0.120	0.039 (-0.011, 0.089)	0.138	0.035 (-0.003, 0.073)	0.097
Country-income groups								
2015	0.017 (0.002, 0.032)	0.093	0.029 (0.019, 0.038)	0.015	0.038 (0.015, 0.061)	0.051	0.031 (0.024, 0.038)	0.007
2016	0.019 (0.005, 0.033)	0.071	0.032 (0.014, 0.049)	0.041	0.042 (0.007, 0.077)	0.087	0.034 (0.019, 0.049)	0.027
2017	0.020 (0.011, 0.028)	0.024	0.032 (0.020, 0.045)	0.021	0.041 (0.013, 0.068)	0.060	0.038 (0.018, 0.058)	0.039
2018	0.020 (0.016, 0.025)	0.007	0.036 (0.019, 0.053)	0.033	0.050 (0.012, 0.087)	0.074	0.038 (0.018, 0.058)	0.039
2019	0.025 (0.002, 0.048)	0.104	0.040 (0.011, 0.068)	0.067	0.046 (0.011, 0.081)	0.074	0.050 (0.012, 0.088)	0.076
2020	0.032 (0.014, 0.050)	0.044	0.056 (0.029, 0.083)	0.033	0.060 (0.036, 0.084)	0.022	0.054 (0.032, 0.075)	0.022
2021	0.038 (0.022, 0.054)	0.026	0.063 (0.023, 0.103)	0.053	0.059 (0.051, 0.067)	0.003	0.065 (0.034, 0.097)	0.032
2022	0.037 (0.011, 0.064)	0.064	0.051 (0.024, 0.077)	0.037	0.065 (0.042, 0.088)	0.018	0.061 (0.033, 0.090)	0.031
2023	0.036 (0.016, 0.056)	0.041	0.053 (0.033, 0.072)	0.019	0.056 (0.024, 0.088)	0.043	0.057 (0.040, 0.075)	0.014

Supplementary Table 6. Economic related-inequalities using GDP per capital in PPP

Vaccine	Year	African Region			Region of the Americas			South-East Asia Region			European Region			Eastern Mediterranean Region			Western Pacific Region		
		n	Concentration index	p value	n	Concentration index	p value	n	Concentration index	p value	n	Concentration index	p value	n	Concentration index	p value	n	Concentration index	p value
DTP1	2015	46	0.024 (-0.002, 0.050)	0.074	33	0.005 (-0.008, 0.017)	0.447	10	0.020 (-0.002, 0.043)	0.088	52	0.008 (-0.002, 0.017)	0.119	19	0.048 (0.018, 0.077)	0.004	25	0.025 (0.006, 0.045)	0.013
	2016	45	0.017 (-0.006, 0.040)	0.156	33	0.002 (-0.007, 0.011)	0.597	10	0.016 (-0.001, 0.033)	0.077	52	0.011 (-0.002, 0.024)	0.111	19	0.057 (0.025, 0.088)	0.002	25	0.021 (0.003, 0.040)	0.023
	2017	45	0.015 (-0.009, 0.038)	0.221	33	0.005 (-0.005, 0.015)	0.288	10	0.011 (-0.002, 0.024)	0.103	52	0.008 (0.000, 0.016)	0.066	20	0.056 (0.028, 0.085)	0.001	25	0.026 (-0.001, 0.053)	0.057
	2018	45	0.013 (-0.009, 0.035)	0.241	33	0.008 (-0.002, 0.018)	0.116	10	0.009 (-0.003, 0.022)	0.150	52	0.003 (-0.002, 0.008)	0.199	20	0.046 (0.015, 0.077)	0.007	25	0.028 (-0.003, 0.059)	0.079
	2019	45	0.012 (-0.010, 0.033)	0.286	33	0.010 (-0.005, 0.025)	0.175	10	0.010 (-0.004, 0.024)	0.151	52	0.003 (-0.002, 0.008)	0.208	20	0.049 (0.018, 0.079)	0.004	25	0.018 (-0.009, 0.045)	0.190
	2020	45	0.010 (-0.013, 0.033)	0.380	33	0.018 (-0.003, 0.040)	0.099	10	0.021 (-0.001, 0.043)	0.074	52	0.009 (0.003, 0.014)	0.002	20	0.055 (0.023, 0.087)	0.002	25	0.019 (-0.005, 0.044)	0.123
	2021	45	0.015 (-0.008, 0.039)	0.198	33	0.029 (0.014, 0.044)	<0.001	10	0.048 (-0.023, 0.119)	0.178	52	0.008 (0.003, 0.013)	0.005	20	0.064 (0.033, 0.096)	<0.001	25	0.030 (-0.002, 0.063)	0.071
	2022	45	0.011 (-0.014, 0.036)	0.381	33	0.029 (0.014, 0.043)	<0.001	10	0.016 (-0.006, 0.038)	0.146	52	0.008 (0.001, 0.015)	0.019	20	0.073 (0.044, 0.101)	<0.001	25	0.023 (-0.002, 0.049)	0.077
	2023	45	0.009 (-0.012, 0.030)	0.404	33	0.029 (0.012, 0.046)	0.001	9	0.013 (-0.018, 0.045)	0.381	51	0.006 (0.000, 0.012)	0.060	18	0.074 (0.038, 0.109)	0.001	24	0.021 (-0.008, 0.049)	0.154
DTP3	2015	46	0.029 (-0.007, 0.066)	0.113	33	0.011 (-0.009, 0.031)	0.276	10	0.029 (-0.001, 0.058)	0.066	52	0.014 (-0.004, 0.031)	0.138	19	0.067 (0.028, 0.106)	0.002	25	0.045 (0.020, 0.071)	0.001
	2016	45	0.022 (-0.011, 0.054)	0.188	33	0.010 (-0.006, 0.026)	0.222	10	0.031 (0.008, 0.055)	0.021	52	0.014 (-0.005, 0.034)	0.141	19	0.072 (0.034, 0.110)	0.001	25	0.048 (0.020, 0.077)	0.002
	2017	45	0.020 (-0.012, 0.052)	0.215	33	0.008 (-0.010, 0.026)	0.385	10	0.027 (0.008, 0.047)	0.016	52	0.011 (-0.002, 0.024)	0.085	20	0.074 (0.037, 0.111)	0.001	25	0.047 (0.015, 0.079)	0.006

	2018	45	0.021 (-0.009, 0.051)	0.173	33	0.013 (-0.002, 0.027)	0.091	10	0.022 (0.002, 0.042)	0.047	52	0.007 (-0.003, 0.016)	0.189	20	0.064 (0.025, 0.103)	0.003	25	0.051 (0.012, 0.090)	0.014
	2019	45	0.022 (-0.008, 0.052)	0.155	33	0.014 (-0.008, 0.037)	0.196	10	0.018 (-0.004, 0.040)	0.121	52	0.004 (-0.004, 0.013)	0.320	20	0.063 (0.025, 0.100)	0.003	25	0.038 (0.004, 0.072)	0.031
	2020	45	0.024 (-0.007, 0.055)	0.121	33	0.027 (-0.003, 0.057)	0.080	10	0.030 (0.001, 0.059)	0.055	52	0.012 (0.003, 0.022)	0.013	20	0.070 (0.030, 0.111)	0.002	25	0.038 (0.009, 0.067)	0.014
	2021	45	0.031 (-0.001, 0.063)	0.056	33	0.037 (0.015, 0.059)	0.002	10	0.056 (-0.030, 0.141)	0.192	52	0.011 (0.002, 0.020)	0.024	20	0.090 (0.050, 0.129)	<0.001	25	0.055 (0.019, 0.091)	0.005
	2022	45	0.026 (-0.007, 0.059)	0.120	33	0.037 (0.015, 0.059)	0.002	10	0.029 (-0.004, 0.062)	0.096	52	0.011 (0.000, 0.021)	0.043	20	0.097 (0.060, 0.134)	<0.001	25	0.052 (0.020, 0.083)	0.003
	2023	45	0.025 (-0.003, 0.053)	0.077	33	0.034 (0.004, 0.064)	0.027	9	0.020 (-0.017, 0.057)	0.278	51	0.008 (-0.002, 0.018)	0.130	18	0.090 (0.049, 0.131)	<0.001	24	0.050 (0.015, 0.084)	0.007
MCV1	2015	46	0.032 (-0.005, 0.068)	0.089	33	0.005 (-0.012, 0.021)	0.566	10	0.042 (0.011, 0.072)	0.017	52	0.007 (-0.006, 0.021)	0.278	19	0.071 (0.037, 0.105)	0.001	25	0.049 (0.026, 0.071)	<0.001
	2016	45	0.024 (-0.013, 0.061)	0.200	33	0.000 (-0.015, 0.015)	0.996	10	0.040 (0.015, 0.065)	0.008	52	0.016 (-0.003, 0.035)	0.107	19	0.070 (0.037, 0.102)	<0.001	25	0.050 (0.022, 0.077)	0.001
	2017	45	0.025 (-0.012, 0.062)	0.186	33	0.009 (-0.008, 0.026)	0.297	10	0.037 (0.018, 0.056)	0.003	52	0.007 (-0.005, 0.020)	0.233	20	0.066 (0.036, 0.096)	<0.001	25	0.048 (0.018, 0.077)	0.003
	2018	45	0.022 (-0.014, 0.058)	0.223	33	0.011 (-0.003, 0.026)	0.121	10	0.027 (0.006, 0.048)	0.024	52	0.009 (-0.006, 0.025)	0.247	20	0.063 (0.030, 0.095)	0.001	25	0.055 (0.016, 0.094)	0.008
	2019	45	0.023 (-0.013, 0.060)	0.211	33	0.008 (-0.012, 0.028)	0.423	10	0.027 (-0.001, 0.056)	0.069	52	0.008 (-0.010, 0.026)	0.362	20	0.066 (0.031, 0.100)	0.001	25	0.040 (0.010, 0.070)	0.011
	2020	45	0.023 (-0.015, 0.060)	0.232	33	0.016 (-0.009, 0.040)	0.215	10	0.024 (-0.011, 0.059)	0.179	52	0.023 (0.003, 0.044)	0.028	20	0.074 (0.036, 0.112)	0.001	25	0.050 (0.022, 0.078)	0.001
	2021	45	0.033 (-0.006, 0.071)	0.095	33	0.033 (0.010, 0.056)	0.007	10	0.060 (-0.013, 0.132)	0.110	52	0.021 (0.000, 0.043)	0.056	20	0.094 (0.056, 0.133)	<0.001	25	0.081 (0.044, 0.118)	<0.001
	2022	45	0.029 (-0.011, 0.068)	0.153	33	0.037 (0.016, 0.058)	0.001	10	0.035 (0.000, 0.070)	0.061	52	0.019 (0.000, 0.039)	0.050	20	0.108 (0.070, 0.146)	<0.001	25	0.052 (0.022, 0.081)	0.002
	2023	45	0.033 (-0.002, 0.067)	0.063	33	0.023 (0.004, 0.042)	0.019	9	0.035 (-0.009, 0.079)	0.125	51	0.015 (-0.007, 0.037)	0.176	18	0.093 (0.054, 0.132)	<0.001	24	0.051 (0.026, 0.076)	<0.001

POL3	2015	46	0.021 (-0.013, 0.055)	0.226	33	0.002 (-0.016, 0.020)	0.805	10	0.031 (0.001, 0.060)	0.053	52	0.010 (-0.003, 0.022)	0.131	19	0.061 (0.025, 0.097)	0.003	25	0.046 (0.022, 0.070)	0.001
	2016	45	0.019 (-0.012, 0.049)	0.231	33	0.005 (-0.014, 0.024)	0.595	10	0.034 (0.011, 0.057)	0.012	52	0.007 (-0.005, 0.018)	0.243	19	0.064 (0.029, 0.099)	0.001	25	0.051 (0.023, 0.078)	0.001
	2017	45	0.018 (-0.013, 0.050)	0.252	33	0.010 (-0.012, 0.032)	0.382	10	0.027 (0.011, 0.044)	0.007	52	0.010 (-0.003, 0.024)	0.125	20	0.065 (0.030, 0.100)	0.001	25	0.050 (0.018, 0.083)	0.004
	2018	45	0.023 (-0.008, 0.055)	0.142	33	0.011 (-0.004, 0.027)	0.139	10	0.022 (0.004, 0.041)	0.032	52	0.006 (-0.003, 0.016)	0.203	20	0.063 (0.026, 0.100)	0.003	25	0.051 (0.016, 0.086)	0.007
	2019	45	0.026 (-0.006, 0.057)	0.107	33	0.014 (-0.007, 0.035)	0.194	10	0.020 (-0.002, 0.041)	0.087	52	0.003 (-0.006, 0.012)	0.472	20	0.055 (0.020, 0.090)	0.004	25	0.038 (0.007, 0.068)	0.019
	2020	45	0.024 (-0.008, 0.056)	0.138	33	0.025 (-0.004, 0.055)	0.089	10	0.029 (-0.002, 0.059)	0.075	52	0.011 (0.002, 0.020)	0.020	20	0.056 (0.016, 0.097)	0.010	25	0.040 (0.012, 0.068)	0.008
	2021	45	0.030 (-0.007, 0.068)	0.112	33	0.038 (0.014, 0.062)	0.003	10	0.052 (-0.025, 0.129)	0.175	52	0.010 (0.000, 0.019)	0.047	20	0.081 (0.044, 0.119)	<0.001	25	0.061 (0.027, 0.096)	0.001
	2022	45	0.032 (-0.004, 0.068)	0.083	33	0.043 (0.018, 0.068)	0.002	10	0.030 (0.003, 0.058)	0.042	52	0.010 (-0.001, 0.021)	0.065	20	0.087 (0.051, 0.124)	<0.001	25	0.053 (0.018, 0.087)	0.005
	2023	45	0.021 (-0.008, 0.050)	0.155	33	0.037 (0.007, 0.067)	0.017	9	0.019 (-0.015, 0.053)	0.261	51	0.005 (-0.004, 0.015)	0.272	18	0.082 (0.045, 0.119)	<0.001	24	0.051 (0.019, 0.082)	0.003

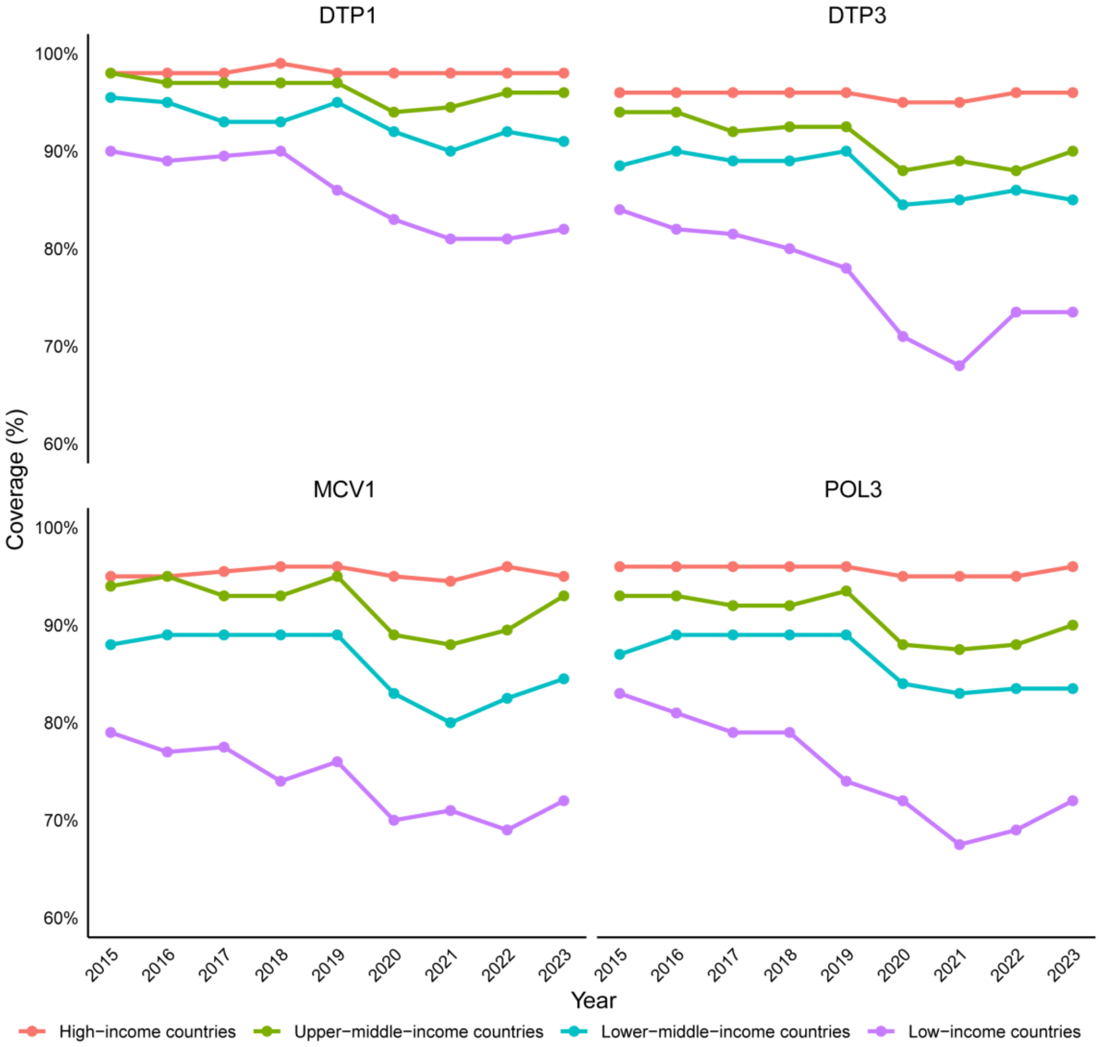

Supplementary Figure 9. Vaccine coverage by country income groupings

Supplementary Table 7. Vaccination coverage in unclassified income countries

Year	Country/territory	DTP1	DTP3	MCV1	POL3
2015	Cook Islands	99	99	99	99
	Niue	99	99	99	99
	occupied Palestinian territory, including east Jerusalem	99	99	99	99
2016	Cook Islands	99	99	90	99
	Niue	99	99	99	99
	occupied Palestinian territory, including east Jerusalem	99	99	99	99
2017	Cook Islands	99	99	98	99
	Niue	99	99	99	99
	occupied Palestinian territory, including east Jerusalem	99	99	99	99
2018	Cook Islands	99	99	99	99
	Niue	99	99	99	99
	occupied Palestinian territory, including east Jerusalem	99	99	99	99
2019	Cook Islands	99	98	99	98
	Niue	99	99	99	99
	occupied Palestinian territory, including east Jerusalem	99	99	99	99
2020	Cook Islands	97	89	87	89
	Niue	99	99	99	99
	occupied Palestinian territory, including east Jerusalem	99	98	99	98
	Venezuela (Bolivarian Republic of)	73	54	76	62
2021	Cook Islands	94	81	74	81
	Niue	99	99	99	99
	occupied Palestinian territory, including east Jerusalem	99	95	98	95
	Venezuela (Bolivarian Republic of)	73	56	68	50
2022	Cook Islands	91	72	61	81
	Niue	99	99	99	99
	occupied Palestinian territory, including east Jerusalem	99	98	97	99
	Venezuela (Bolivarian Republic of)	56	43	52	47
2023	Cook Islands	95	86	84	69
	Niue	99	99	99	99
	occupied Palestinian territory, including east Jerusalem	88	88	89	89
	Venezuela (Bolivarian Republic of)	65	54	68	61

Supplementary Table 8. Equalities between country-income groups in vaccination coverage

Type	Year	DTP1		DTP3		MCV1		POL3	
		**concentration index**	***p* value**	**concentration index**	***p* value**	**concentration index**	***p* value**	**concentration index**	***p* value**
Economic-related	2015	0.017 (0.002, 0.032)	0.093	0.029 (0.019, 0.038)	0.015	0.038 (0.015, 0.061)	0.051	0.031 (0.024, 0.038)	0.007
	2016	0.019 (0.005, 0.033)	0.071	0.032 (0.014, 0.049)	0.041	0.042 (0.007, 0.077)	0.087	0.034 (0.019, 0.049)	0.027
	2017	0.020 (0.011, 0.028)	0.024	0.032 (0.020, 0.045)	0.021	0.041 (0.013, 0.068)	0.060	0.038 (0.018, 0.058)	0.039
	2018	0.020 (0.016, 0.025)	0.007	0.036 (0.019, 0.053)	0.033	0.050 (0.012, 0.087)	0.074	0.038 (0.018, 0.058)	0.039
	2019	0.025 (0.002, 0.048)	0.104	0.040 (0.011, 0.068)	0.067	0.046 (0.011, 0.081)	0.074	0.050 (0.012, 0.088)	0.076
	2020	0.032 (0.014, 0.050)	0.044	0.056 (0.029, 0.083)	0.033	0.060 (0.036, 0.084)	0.022	0.054 (0.032, 0.075)	0.022
	2021	0.038 (0.022, 0.054)	0.026	0.063 (0.023, 0.103)	0.053	0.059 (0.051, 0.067)	0.003	0.065 (0.034, 0.097)	0.032
	2022	0.037 (0.011, 0.064)	0.064	0.051 (0.024, 0.077)	0.037	0.065 (0.042, 0.088)	0.018	0.061 (0.033, 0.090)	0.031
	2023	0.036 (0.016, 0.056)	0.041	0.053 (0.033, 0.072)	0.019	0.056 (0.024, 0.088)	0.043	0.057 (0.040, 0.075)	0.014
Education-related	2015	0.017 (0.002, 0.032)	0.093	0.029 (0.019, 0.038)	0.015	0.038 (0.015, 0.061)	0.051	0.031 (0.024, 0.038)	0.007
	2016	0.019 (0.005, 0.033)	0.071	0.032 (0.014, 0.049)	0.041	0.042 (0.007, 0.077)	0.087	0.034 (0.019, 0.049)	0.027
	2017	0.020 (0.011, 0.028)	0.024	0.032 (0.020, 0.045)	0.021	0.041 (0.013, 0.068)	0.060	0.038 (0.018, 0.058)	0.039
	2018	0.020 (0.016, 0.025)	0.007	0.036 (0.019, 0.053)	0.033	0.050 (0.012, 0.087)	0.074	0.038 (0.018, 0.058)	0.039
	2019	0.025 (0.002, 0.048)	0.104	0.040 (0.011, 0.068)	0.067	0.046 (0.011, 0.081)	0.074	0.050 (0.012, 0.088)	0.076
	2020	0.032 (0.014, 0.050)	0.044	0.056 (0.029, 0.083)	0.033	0.060 (0.036, 0.084)	0.022	0.054 (0.032, 0.075)	0.022
	2021	0.038 (0.022, 0.054)	0.026	0.063 (0.023, 0.103)	0.053	0.059 (0.051, 0.067)	0.003	0.065 (0.034, 0.097)	0.032
	2022	0.037 (0.011, 0.064)	0.064	0.051 (0.024, 0.077)	0.037	0.065 (0.042, 0.088)	0.018	0.061 (0.033, 0.090)	0.031

Supplementary Table 9. Income-related equalities in vaccination coverage by country-income group

Type	Year	Low-income countries			Lower middle-income countries			Upper middle-income countries			High-income countries		
		n	Concentration index	*p* value	n	Concentration index	*p* value	n	Concentration index	*p* value	n	Concentration index	*p* value
DTP1	2015	29	0.018 (-0.020, 0.056)	0.338	50	0.014 (-0.006, 0.035)	0.174	54	0.002 (-0.010, 0.015)	0.707	56	-0.001 (-0.005, 0.002)	0.357
	2016	28	0.031 (-0.005, 0.067)	0.087	51	0.023 (0.002, 0.044)	0.035	54	0.009 (-0.001, 0.018)	0.072	55	-0.001 (-0.004, 0.002)	0.382
	2017	31	0.022 (-0.013, 0.058)	0.216	45	0.008 (-0.013, 0.030)	0.456	54	0.003 (-0.007, 0.012)	0.548	58	0.001 (-0.003, 0.004)	0.615
	2018	28	0.025 (-0.012, 0.061)	0.187	46	0.000 (-0.020, 0.019)	0.982	57	0.000 (-0.012, 0.011)	0.966	57	-0.001 (-0.004, 0.002)	0.496
	2019	26	0.017 (-0.022, 0.057)	0.385	49	0.005 (-0.014, 0.024)	0.597	53	-0.001 (-0.013, 0.010)	0.799	60	-0.001 (-0.005, 0.002)	0.483
	2020	24	0.019 (-0.022, 0.060)	0.345	54	0.012 (-0.006, 0.030)	0.189	53	0.010 (-0.006, 0.026)	0.205	57	-0.001 (-0.005, 0.003)	0.625
	2021	25	0.033 (-0.007, 0.073)	0.103	53	0.017 (-0.007, 0.041)	0.159	51	0.010 (-0.004, 0.023)	0.153	58	0.002 (-0.002, 0.007)	0.355
	2022	23	0.027 (-0.013, 0.067)	0.176	54	0.008 (-0.013, 0.029)	0.453	51	0.008 (-0.005, 0.022)	0.226	59	0.000 (-0.004, 0.005)	0.865
	2023	22	0.035 (-0.011, 0.081)	0.138	48	0.008 (-0.014, 0.030)	0.449	51	0.012 (-0.002, 0.025)	0.085	61	0.002 (-0.002, 0.007)	0.333
DTP3	2015	29	0.020 (-0.032, 0.073)	0.437	50	0.017 (-0.014, 0.048)	0.282	54	0.001 (-0.018, 0.021)	0.882	56	-0.001 (-0.006, 0.003)	0.564
	2016	28	0.033 (-0.015, 0.082)	0.173	51	0.026 (-0.004, 0.057)	0.089	54	0.013 (-0.003, 0.029)	0.121	55	-0.004 (-0.009, 0.001)	0.102
	2017	31	0.025 (-0.022, 0.072)	0.284	45	0.008 (-0.021, 0.037)	0.574	54	0.002 (-0.015, 0.019)	0.811	58	0.002 (-0.004, 0.008)	0.416
	2018	28	0.024 (-0.027, 0.075)	0.351	46	-0.001 (-0.026, 0.024)	0.952	57	0.006 (-0.012, 0.024)	0.492	57	-0.001 (-0.006, 0.004)	0.746
	2019	26	0.015 (-0.041, 0.071)	0.593	49	0.007 (-0.017, 0.030)	0.568	53	0.006 (-0.011, 0.023)	0.493	60	0.000 (-0.005, 0.005)	1.000
	2020	24	0.030 (-0.025, 0.085)	0.276	54	0.016 (-0.007, 0.040)	0.176	53	0.014 (-0.007, 0.036)	0.191	57	-0.001 (-0.008, 0.005)	0.650
	2021	25	0.049 (-0.005, 0.103)	0.075	53	0.017 (-0.014, 0.049)	0.273	51	0.012 (-0.005, 0.029)	0.152	58	0.006 (-0.001, 0.012)	0.093
	2022	23	0.047 (-0.006, 0.100)	0.083	54	0.012 (-0.017, 0.041)	0.418	51	0.010 (-0.008, 0.027)	0.279	59	0.002 (-0.004, 0.009)	0.514
	2023	22	0.051 (-0.006, 0.107)	0.078	48	0.011 (-0.018, 0.041)	0.441	51	0.007 (-0.012, 0.027)	0.459	61	0.002 (-0.007, 0.011)	0.657
MCV1	2015	29	0.017 (-0.032, 0.066)	0.488	50	0.019 (-0.007, 0.045)	0.156	54	0.014 (-0.007, 0.034)	0.184	56	-0.004 (-0.009, 0.002)	0.181
	2016	28	0.035 (-0.014, 0.083)	0.157	51	0.029 (0.001, 0.057)	0.045	54	0.015 (-0.006, 0.035)	0.153	55	-0.002 (-0.009, 0.004)	0.477
	2017	31	0.034 (-0.013, 0.081)	0.153	45	0.018 (-0.010, 0.046)	0.198	54	0.001 (-0.019, 0.021)	0.954	58	-0.001 (-0.007, 0.004)	0.603
	2018	28	0.029 (-0.022, 0.081)	0.259	46	0.007 (-0.020, 0.034)	0.593	57	0.009 (-0.015, 0.033)	0.445	57	0.000 (-0.006, 0.005)	0.905
	2019	26	0.030 (-0.026, 0.087)	0.285	49	0.016 (-0.011, 0.043)	0.237	53	-0.002 (-0.027, 0.022)	0.851	60	-0.003 (-0.009, 0.003)	0.274
	2020	24	0.025 (-0.033, 0.084)	0.390	54	0.011 (-0.015, 0.038)	0.390	53	0.011 (-0.018, 0.039)	0.465	57	0.000 (-0.006, 0.006)	0.966
	2021	25	0.047 (-0.011, 0.104)	0.112	53	0.026 (-0.008, 0.060)	0.130	51	0.009 (-0.020, 0.039)	0.526	58	0.005 (-0.003, 0.012)	0.200

	2022	23	0.048 (-0.012, 0.107)	0.114	54	0.016 (-0.015, 0.046)	0.319	51	0.010 (-0.017, 0.038)	0.456	59	0.001 (-0.006, 0.008)	0.743
	2023	22	0.055 (-0.006, 0.116)	0.076	48	0.023 (-0.006, 0.051)	0.120	51	-0.009 (-0.035, 0.017)	0.494	61	0.003 (-0.005, 0.010)	0.507
POL3	2015	29	0.013 (-0.032, 0.058)	0.573	50	0.018 (-0.009, 0.045)	0.181	54	0.002 (-0.018, 0.021)	0.856	56	0.002 (-0.003, 0.007)	0.473
	2016	28	0.024 (-0.019, 0.068)	0.266	51	0.029 (0.003, 0.054)	0.029	54	0.010 (-0.007, 0.027)	0.226	55	-0.001 (-0.007, 0.005)	0.741
	2017	31	0.018 (-0.026, 0.062)	0.406	45	0.009 (-0.021, 0.038)	0.570	54	0.002 (-0.016, 0.021)	0.805	58	0.003 (-0.003, 0.009)	0.278
	2018	28	0.011 (-0.039, 0.061)	0.657	46	0.005 (-0.019, 0.028)	0.694	57	0.008 (-0.011, 0.027)	0.401	57	0.000 (-0.006, 0.006)	0.998
	2019	26	0.008 (-0.048, 0.063)	0.782	49	0.011 (-0.012, 0.034)	0.349	53	0.008 (-0.009, 0.025)	0.348	60	0.001 (-0.005, 0.007)	0.727
	2020	24	0.018 (-0.035, 0.071)	0.492	54	0.022 (-0.003, 0.046)	0.088	53	0.014 (-0.007, 0.036)	0.179	57	0.000 (-0.007, 0.007)	0.980
	2021	25	0.022 (-0.041, 0.085)	0.487	53	0.019 (-0.013, 0.051)	0.250	51	0.017 (-0.001, 0.036)	0.069	58	0.007 (0.000, 0.013)	0.060
	2022	23	0.022 (-0.034, 0.078)	0.434	54	0.018 (-0.013, 0.050)	0.255	51	0.013 (-0.007, 0.032)	0.213	59	0.004 (-0.003, 0.011)	0.247
	2023	22	0.039 (-0.013, 0.092)	0.140	48	0.012 (-0.018, 0.042)	0.435	51	0.015 (-0.005, 0.035)	0.133	61	0.004 (-0.005, 0.013)	0.357

n represents the number of countries/territories included in the analysis.

Supplementary Table 10. Education-related equalities in vaccination coverage by country-income group

Type	Year	Low-income countries			Lower middle-income countries			Upper middle-income countries			High-income countries		
		n	Concentration index	P value	n	Concentration index	P value	n	Concentration index	P value	n	Concentration index	P value
DTP1	2015	29	0.004 (-0.031, 0.040)	0.799	50	0.013 (-0.007, 0.034)	0.203	55	0.013 (0.002, 0.025)	0.025	53	-0.004 (-0.006, -0.002)	0.001
	2016	29	0.006 (-0.031, 0.043)	0.756	51	0.018 (-0.004, 0.039)	0.107	54	0.007 (-0.002, 0.017)	0.145	53	-0.003 (-0.006, -0.001)	0.010
	2017	32	0.014 (-0.020, 0.048)	0.420	45	0.016 (-0.005, 0.037)	0.130	54	0.004 (-0.005, 0.014)	0.341	56	0.000 (-0.003, 0.003)	0.938
	2018	29	0.018 (-0.015, 0.052)	0.282	46	0.015 (-0.004, 0.034)	0.128	57	0.006 (-0.006, 0.017)	0.322	56	-0.002 (-0.005, 0.001)	0.161
	2019	27	0.013 (-0.022, 0.049)	0.448	49	0.020 (0.002, 0.038)	0.031	54	0.012 (0.001, 0.023)	0.035	58	-0.002 (-0.005, 0.002)	0.298
	2020	25	0.008 (-0.029, 0.044)	0.669	54	0.026 (0.009, 0.043)	0.004	53	0.011 (-0.005, 0.027)	0.163	55	-0.001 (-0.005, 0.003)	0.652
	2021	26	0.010 (-0.027, 0.047)	0.575	53	0.027 (0.004, 0.050)	0.024	52	0.019 (0.007, 0.031)	0.003	56	-0.001 (-0.005, 0.004)	0.833
	2022	25	0.015 (-0.023, 0.054)	0.423	54	0.024 (0.003, 0.044)	0.024	52	0.013 (-0.001, 0.026)	0.065	58	-0.002 (-0.006, 0.002)	0.385
DTP3	2015	29	0.007 (-0.042, 0.057)	0.773	50	0.021 (-0.010, 0.051)	0.183	55	0.022 (0.004, 0.040)	0.018	53	-0.007 (-0.010, -0.004)	<0.001
	2016	29	0.008 (-0.040, 0.056)	0.747	51	0.028 (-0.003, 0.058)	0.074	54	0.007 (-0.010, 0.023)	0.412	53	-0.007 (-0.011, -0.003)	0.001
	2017	32	0.019 (-0.025, 0.064)	0.386	45	0.022 (-0.006, 0.050)	0.117	54	0.010 (-0.007, 0.028)	0.240	56	-0.002 (-0.008, 0.004)	0.431
	2018	29	0.026 (-0.020, 0.073)	0.257	46	0.019 (-0.005, 0.043)	0.117	57	0.006 (-0.013, 0.025)	0.506	56	-0.004 (-0.009, 0.001)	0.143
	2019	27	0.016 (-0.035, 0.066)	0.539	49	0.027 (0.005, 0.049)	0.020	54	0.012 (-0.005, 0.030)	0.170	58	-0.002 (-0.008, 0.003)	0.414
	2020	25	0.017 (-0.033, 0.067)	0.496	54	0.034 (0.011, 0.056)	0.004	53	0.017 (-0.005, 0.038)	0.119	55	-0.002 (-0.008, 0.004)	0.514
	2021	26	0.021 (-0.030, 0.073)	0.410	53	0.036 (0.006, 0.066)	0.022	52	0.023 (0.008, 0.039)	0.004	56	-0.001 (-0.008, 0.006)	0.857
	2022	25	0.021 (-0.032, 0.074)	0.434	54	0.036 (0.008, 0.064)	0.012	52	0.015 (-0.002, 0.032)	0.083	58	-0.004 (-0.011, 0.002)	0.202
MCV1	2015	29	0.012 (-0.036, 0.059)	0.622	50	0.036 (0.011, 0.061)	0.006	55	0.017 (-0.003, 0.036)	0.097	53	-0.003 (-0.008, 0.002)	0.285
	2016	29	0.011 (-0.037, 0.059)	0.647	51	0.038 (0.011, 0.066)	0.007	54	0.002 (-0.019, 0.023)	0.842	53	-0.005 (-0.010, 0.001)	0.089
	2017	32	0.030 (-0.015, 0.075)	0.188	45	0.038 (0.012, 0.064)	0.006	54	0.012 (-0.008, 0.032)	0.223	56	-0.005 (-0.010, 0.000)	0.041
	2018	29	0.036 (-0.011, 0.083)	0.135	46	0.032 (0.007, 0.057)	0.014	57	0.007 (-0.017, 0.031)	0.546	56	-0.005 (-0.011, 0.000)	0.062
	2019	27	0.027 (-0.026, 0.080)	0.306	49	0.043 (0.019, 0.067)	0.001	54	0.011 (-0.012, 0.035)	0.336	58	-0.005 (-0.011, 0.000)	0.064
	2020	25	0.018 (-0.037, 0.073)	0.506	54	0.043 (0.020, 0.067)	0.001	53	0.004 (-0.025, 0.032)	0.799	55	-0.001 (-0.006, 0.005)	0.846
	2021	26	0.021 (-0.036, 0.077)	0.464	53	0.051 (0.019, 0.082)	0.002	52	0.014 (-0.015, 0.043)	0.345	56	0.001 (-0.007, 0.008)	0.899
	2022	25	0.026 (-0.032, 0.084)	0.370	54	0.049 (0.021, 0.077)	0.001	52	0.010 (-0.018, 0.037)	0.482	58	-0.005 (-0.012, 0.002)	0.200
POL3	2015	29	0.007 (-0.036, 0.049)	0.750	50	0.028 (0.002, 0.054)	0.036	55	0.020 (0.002, 0.039)	0.032	53	-0.005 (-0.010, 0.000)	0.044

2016	29	0.003 (-0.041, 0.047)	0.883	51	0.038 (0.013, 0.062)	0.003	54	0.008 (-0.009, 0.025)	0.358	53	-0.006 (-0.012, -0.001)	0.020
2017	32	0.021 (-0.020, 0.062)	0.299	45	0.025 (-0.004, 0.054)	0.095	54	0.013 (-0.005, 0.031)	0.166	56	-0.004 (-0.010, 0.002)	0.215
2018	29	0.021 (-0.025, 0.067)	0.368	46	0.023 (0.000, 0.046)	0.048	57	0.009 (-0.012, 0.029)	0.395	56	-0.006 (-0.012, -0.001)	0.026
2019	27	0.019 (-0.033, 0.070)	0.465	49	0.029 (0.008, 0.051)	0.009	54	0.011 (-0.007, 0.028)	0.230	58	-0.004 (-0.010, 0.001)	0.128
2020	25	0.017 (-0.032, 0.065)	0.496	54	0.037 (0.013, 0.060)	0.003	53	0.018 (-0.003, 0.039)	0.097	55	-0.004 (-0.011, 0.004)	0.341
2021	26	0.022 (-0.037, 0.080)	0.464	53	0.039 (0.008, 0.069)	0.015	52	0.030 (0.012, 0.047)	0.001	56	-0.002 (-0.009, 0.005)	0.553
2022	25	0.012 (-0.042, 0.066)	0.646	54	0.036 (0.006, 0.067)	0.020	52	0.018 (-0.001, 0.038)	0.061	58	-0.005 (-0.012, 0.002)	0.145

n represents the number of countries/territories included in the analysis.

Supplementary Table 11. Stock-outs of vaccine by country-income group

Vaccine	Year	Variable	Low-income countries	Lower middle-income countries	Upper middle-income countries	High-income countries	Unclassified countries
DTPCV	2015	Yes	2 (6.45)	8 (16.00)	15 (27.27)	6 (8.70)	1 (12.50)
		No	25 (80.65)	40 (80.00)	32 (58.18)	43 (62.32)	3 (37.50)
		NR/ND	0 (0.00)	0 (0.00)	0 (0.00)	2 (2.90)	0 (0.00)
		No data	4 (12.90)	2 (4.00)	8 (14.55)	18 (26.09)	4 (50.00)
	2016	Yes	1 (3.23)	5 (9.80)	11 (20.00)	8 (11.76)	0 (0.00)
		No	28 (90.32)	42 (82.35)	38 (69.09)	41 (60.29)	3 (37.50)
		NR/ND	1 (3.23)	2 (3.92)	0 (0.00)	1 (1.47)	0 (0.00)
		No data	1 (3.23)	2 (3.92)	6 (10.91)	18 (26.47)	5 (62.50)
	2017	Yes	1 (2.94)	1 (2.22)	7 (12.73)	8 (11.27)	1 (12.50)
		No	32 (94.12)	42 (93.33)	42 (76.36)	55 (77.46)	6 (75.00)
		NR/ND	1 (2.94)	1 (2.22)	2 (3.64)	2 (2.82)	0 (0.00)
		No data	0 (0.00)	1 (2.22)	4 (7.27)	6 (8.45)	1 (12.50)
	2018	Yes	3 (9.68)	4 (8.89)	8 (13.56)	7 (10.00)	0 (0.00)
		No	25 (80.65)	38 (84.44)	46 (77.97)	56 (80.00)	7 (87.50)
		NR/ND	1 (3.23)	1 (2.22)	2 (3.39)	1 (1.43)	0 (0.00)
		No data	2 (6.45)	2 (4.44)	3 (5.08)	6 (8.57)	1 (12.50)
	2019	Yes	0 (0.00)	3 (6.12)	10 (18.18)	7 (9.59)	0 (0.00)
		No	28 (96.55)	42 (87.76)	39 (70.91)	53 (72.6)	0 (0.00)
		NR/ND	0 (0.00)	1 (2.04)	0 (0.00)	0 (0.00)	0 (0.00)
		No data	1 (3.45)	2 (4.08)	6 (10.91)	13 (17.81)	8 (100.00)
	2020	Yes	5 (18.52)	8 (15.09)	7 (12.96)	4 (5.71)	1 (11.11)
		No	20 (74.07)	40 (75.47)	38 (70.37)	50 (71.43)	3 (33.33)
		NR/ND	0 (0.00)	2 (3.77)	1 (1.85)	5 (7.14)	0 (0.00)
		No data	2 (7.41)	3 (5.66)	8 (14.81)	11 (15.71)	5 (55.56)
	2021	Yes	4 (14.29)	3 (5.77)	5 (9.43)	6 (8.45)	0 (0.00)
		No	22 (78.57)	45 (86.54)	38 (71.70)	46 (64.79)	6 (66.67)
		NR/ND	0 (0.00)	1 (1.92)	0 (0.00)	7 (9.86)	0 (0.00)
		No data	2 (7.14)	3 (5.77)	10 (18.87)	12 (16.9)	3 (33.33)
	2022	Yes	5 (19.23)	8 (15.09)	6 (11.32)	6 (8.33)	1 (11.11)
		No	19 (73.08)	42 (79.25)	41 (77.36)	55 (76.39)	4 (44.44)
		NR/ND	0 (0.00)	0 (0.00)	0 (0.00)	3 (4.17)	0 (0.00)
		No data	2 (7.69)	3 (5.66)	6 (11.32)	8 (11.11)	4 (44.44)
MCV	2015	Yes	1 (3.23)	5 (10.00)	5 (9.09)	2 (2.90)	0 (0.00)
		No	27 (87.10)	42 (84.00)	43 (78.18)	43 (62.32)	4 (50.00)
		NR/ND	0 (0.00)	2 (4.00)	0 (0.00)	3 (4.35)	0 (0.00)
		No data	3 (9.68)	1 (2.00)	7 (12.73)	21 (30.43)	4 (50.00)
	2016	Yes	1 (3.23)	4 (7.84)	4 (7.27)	3 (4.41)	0 (0.00)
		No	29 (93.55)	45 (88.24)	44 (80.00)	45 (66.18)	3 (37.50)
		NR/ND	0 (0.00)	0 (0.00)	0 (0.00)	1 (1.47)	0 (0.00)
		No data	1 (3.23)	2 (3.92)	7 (12.73)	19 (27.94)	5 (62.50)
	2017	Yes	2 (5.88)	4 (8.89)	5 (9.09)	5 (7.04)	0 (0.00)
		No	30 (88.24)	38 (84.44)	46 (83.64)	57 (80.28)	6 (75.00)
		NR/ND	2 (5.88)	0 (0.00)	0 (0.00)	1 (1.41)	0 (0.00)
		No data	0 (0.00)	3 (6.67)	4 (7.27)	8 (11.27)	2 (25.00)
	2018	Yes	4 (12.90)	9 (20.00)	8 (13.56)	4 (5.71)	0 (0.00)
		No	26 (83.87)	33 (73.33)	46 (77.97)	58 (82.86)	7 (87.50)

		NR/ND	0 (0.00)	1 (2.22)	1 (1.69)	1 (1.43)	0 (0.00)
		No data	1 (3.23)	2 (4.44)	4 (6.78)	7 (10.00)	1 (12.50)
	2019	Yes	2 (6.90)	6 (12.50)	9 (16.36)	5 (6.85)	1 (12.50)
		No	26 (89.66)	40 (83.33)	40 (72.73)	54 (73.97)	7 (87.50)
		NR/ND	1 (3.45)	1 (2.08)	1 (1.82)	0 (0.00)	0 (0.00)
		No data	0 (0.00)	1 (2.08)	5 (9.09)	14 (19.18)	0 (0.00)
	2020	Yes	0 (0.00)	7 (13.21)	6 (11.11)	3 (4.29)	0 (0.00)
		No	24 (88.89)	42 (79.25)	38 (70.37)	49 (70.00)	4 (44.44)
		NR/ND	1 (3.70)	1 (1.89)	0 (0.00)	5 (7.14)	0 (0.00)
		No data	2 (7.41)	3 (5.66)	11 (18.52)	13 (18.57)	5 (55.56)
	2021	Yes	4 (14.29)	3 (5.77)	5 (9.43)	3 (4.23)	0 (0.00)
		No	20 (71.43)	42 (80.77)	38 (71.70)	49 (69.01)	5 (55.56)
		NR/ND	1 (3.57)	3 (5.77)	0 (0.00)	6 (8.45)	0 (0.00)
		No data	3 (10.71)	4 (7.69)	10 (18.87)	13 (18.31)	4 (44.44)
	2022	Yes	5 (19.23)	6 (11.32)	8 (15.09)	5 (6.94)	1 (11.11)
		No	17 (65.38)	41 (77.36)	36 (67.92)	55 (76.39)	4 (44.44)
		NR/ND	0 (0.00)	2 (3.77)	0 (0.00)	4 (5.56)	0 (0.00)
		No data	4 (15.38)	4 (7.55)	9 (16.98)	8 (11.11)	4 (44.44)
IPV	2015	Yes	1 (3.23)	2 (4.00)	3 (5.45)	0 (0.00)	1 (12.50)
		No	16 (51.61)	30 (60.00)	35 (63.64)	44 (63.77)	2 (25.00)
		NR/ND	11 (35.48)	10 (20.00)	6 (10.91)	3 (4.35)	1 (12.50)
		No data	3 (9.68)	8 (16.00)	11 (20.00)	22 (31.88)	4 (50.00)
	2016	Yes	10 (32.26)	14 (27.45)	9 (16.36)	2 (2.94)	0 (0.00)
		No	11 (35.48)	27 (52.94)	38 (69.09)	45 (66.18)	3 (37.5)
		NR/ND	9 (29.03)	5 (9.80)	1 (1.82)	2 (2.94)	0 (0.00)
		No data	1 (3.23)	5 (9.80)	7 (12.73)	19 (27.94)	5 (62.5)
	2017	Yes	12 (35.29)	14 (31.11)	12 (21.82)	9 (12.68)	1 (12.5)
		No	13 (38.24)	25 (54.35)	37 (67.27)	52 (73.24)	5 (62.5)
		NR/ND	9 (26.47)	2 (4.44)	2 (3.64)	3 (4.23)	0 (0.00)
		No data	0 (0.00)	4 (8.89)	4 (7.27)	7 (9.86)	2 (25.00)
	2018	Yes	7 (22.58)	6 (13.33)	6 (10.17)	5 (7.14)	0 (0.00)
		No	21 (67.74)	34 (75.56)	46 (77.97)	53 (75.71)	7 (87.50)
		NR/ND	1 (3.23)	2 (4.44)	2 (3.39)	2 (2.86)	0 (0.00)
		No data	2 (6.45)	3 (6.67)	5 (8.47)	10 (14.29)	1 (12.50)
	2019	Yes	3 (10.34)	3 (6.25)	7 (12.73)	2 (2.74)	0 (0.00)
		No	25 (86.21)	42 (87.5)	40 (72.73)	53 (72.6)	8 (100)
		NR/ND	1 (3.45)	1 (2.08)	2 (3.64)	3 (4.11)	0 (0.00)
		No data	0 (0.00)	2 (4.17)	6 (10.91)	15 (20.55)	0 (0.00)
	2020	Yes	4 (14.81)	9 (16.98)	3 (5.56)	2 (2.86)	0 (0.00)
		No	21 (77.78)	40 (75.47)	40 (74.07)	47 (67.14)	4 (44.44)
		NR/ND	0 (0.00)	1 (1.89)	1 (1.85)	8 (11.43)	0 (0.00)
		No data	2 (7.41)	3 (5.66)	10 (18.52)	13 (18.57)	5 (55.56)
	2021	Yes	4 (14.29)	6 (11.54)	4 (7.55)	3 (4.23)	0 (0.00)
		No	22 (78.57)	41 (78.85)	34 (64.15)	45 (63.38)	5 (55.56)
		NR/ND	0 (0.00)	2 (3.85)	5 (9.43)	10 (14.08)	0 (0.00)
		No data	2 (7.14)	3 (5.77)	10 (18.87)	13 (18.31)	4 (44.44)
	2022	Yes	1 (3.85)	6 (11.32)	5 (9.43)	5 (6.94)	1 (11.11)
		No	21 (80.77)	43 (81.13)	37 (69.81)	52 (72.22)	4 (44.44)
		NR/ND	1 (3.85)	0 (0.00)	3 (5.66)	6 (8.33)	0 (0.00)
		No data	3 (11.54)	4 (7.55)	8 (15.09)	9 (12.5)	4 (44.44)

Supplementary Table 12. Stock-outs of home-based vaccination records by country-income group

Year	Variable	Low-income countries	Lower middle-income countries	Upper middle-income countries	High-income countries	Unclassified countries
2015	Yes	7 (22.58)	10 (20.00)	6 (10.91)	0 (0.00)	0 (0.00)
	No	22 (70.97)	30 (60.00)	36 (65.45)	32 (46.38)	4 (50.00)
	NR/ND	1 (3.23)	7 (14.00)	10 (18.18)	13 (18.84)	0 (0.00)
	No data	1 (3.23)	3 (6.00)	3 (5.45)	24 (34.78)	4 (50.00)
2016	Yes	10 (32.26)	12 (23.53)	6 (10.91)	2 (2.94)	0 (0.00)
	No	17 (54.84)	27 (52.94)	34 (61.82)	30 (44.12)	3 (37.50)
	NR/ND	3 (9.68)	9 (17.65)	12 (21.82)	13 (19.12)	0 (0.00)
	No data	1 (3.23)	3 (5.88)	3 (5.45)	23 (33.82)	5 (62.50)
2017	Yes	10 (29.41)	14 (31.11)	3 (5.45)	2 (2.82)	1 (12.50)
	No	20 (58.82)	21 (46.67)	33 (60.00)	43 (60.56)	7 (87.50)
	NR/ND	4 (11.76)	7 (15.56)	17 (30.91)	16 (22.54)	0 (0.00)
	No data	0 (0.00)	3 (6.67)	2 (3.64)	10 (14.08)	0 (0.00)
2018	Yes	12 (38.71)	11 (24.44)	8 (13.56)	0 (0.00)	1 (12.50)
	No	13 (41.94)	24 (53.33)	31 (52.54)	46 (65.71)	4 (50.00)
	NR/ND	4 (12.90)	6 (13.33)	18 (30.51)	16 (22.86)	1 (12.50)
	No data	2 (6.45)	4 (8.89)	2 (3.39)	8 (11.43)	2 (25.00)
2019	Yes	8 (27.59)	10 (20.83)	5 (9.09)	4 (5.48)	1 (12.50)
	No	18 (62.07)	30 (62.5)	33 (60.00)	40 (54.79)	5 (62.50)
	NR/ND	3 (10.34)	6 (12.5)	11 (20.00)	12 (16.44)	2 (25.00)
	No data	0 (0.00)	2 (4.17)	6 (10.91)	17 (23.29)	0 (0.00)
2020	Yes	8 (29.63)	10 (18.87)	5 (9.26)	2 (2.86)	0 (0.00)
	No	17 (62.96)	35 (66.04)	33 (61.11)	40 (57.14)	2 (22.22)
	NR/ND	0 (0.00)	4 (7.55)	10 (18.52)	11 (15.71)	0 (0.00)
	No data	2 (7.41)	4 (7.55)	6 (11.11)	17 (24.29)	7 (77.78)
2021	Yes	8 (28.57)	6 (11.54)	5 (9.43)	0 (0.00)	0 (0.00)
	No	15 (53.57)	34 (65.38)	28 (52.83)	39 (54.93)	6 (66.67)
	NR/ND	3 (10.71)	8 (15.38)	8 (15.09)	13 (18.31)	0 (0.00)
	No data	2 (7.14)	4 (7.69)	12 (22.64)	19 (26.76)	3 (33.33)
2022	Yes	10 (38.46)	6 (11.32)	7 (13.21)	4 (5.56)	0 (0.00)
	No	13 (50.00)	36 (67.92)	31 (58.49)	39 (54.17)	5 (55.56)
	NR/ND	1 (3.85)	9 (16.98)	9 (16.98)	15 (20.83)	0 (0.00)
	No data	2 (7.69)	2 (3.77)	6 (11.32)	14 (19.44)	4 (44.44)
2023	Yes	9 (34.62)	7 (14.29)	5 (9.26)	1 (1.33)	0 (0.00)
	No	10 (38.46)	35 (71.43)	36 (66.67)	42 (56.00)	6 (66.67)
	NR/ND	4 (15.38)	4 (8.16)	8 (14.81)	23 (30.67)	0 (0.00)
	No data	3 (11.54)	3 (6.12)	5 (9.26)	9 (12.00)	3 (33.33)

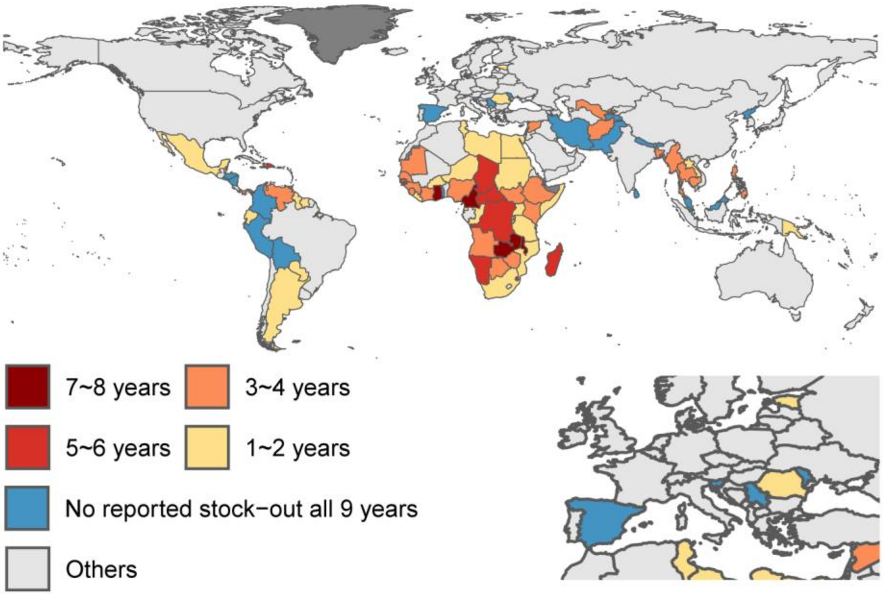

Supplementary Figure 10. The reported frequency of national-level stock-outs of home-based vaccination records

Base map data from Natural Earth (public domain), rendered using R packages rnaturalearth, rnaturalearthdata, and sf.

Supplementary Table 13. Participants' characteristics

Variables	Lower vaccine confidence (n=28,090)	High vaccine confidence (n=94,056)
Age group		
15 to 29 years	8049 (22.3)	27,994 (77.7)
30 to 49 years	10,136 (22.6)	34,787 (77.4)
≥50 years	9905 (24.1)	31,275 (75.9)
Sex		
Male	13,044 (23.1)	43,371 (76.9)
Female	15,046 (22.9)	50,685 (77.1)
Education level		
Primary or below	5448 (15.4)	29,819 (84.6)
Secondary	16,006 (25.3)	47,348 (74.7)
Tertiary or above	6636 (28.2)	16,889 (71.8)
Household Income		
Poorest 20%	4452 (22.7)	15,181 (77.3)
Second 20%	4932 (22.8)	16,715 (77.2)
Middle 20%	5416 (22.8)	18,378 (77.2)
Fourth 20%	5883 (22.7)	20,067 (77.3)
Top 20%	7407 (23.8)	23,715 (76.2)

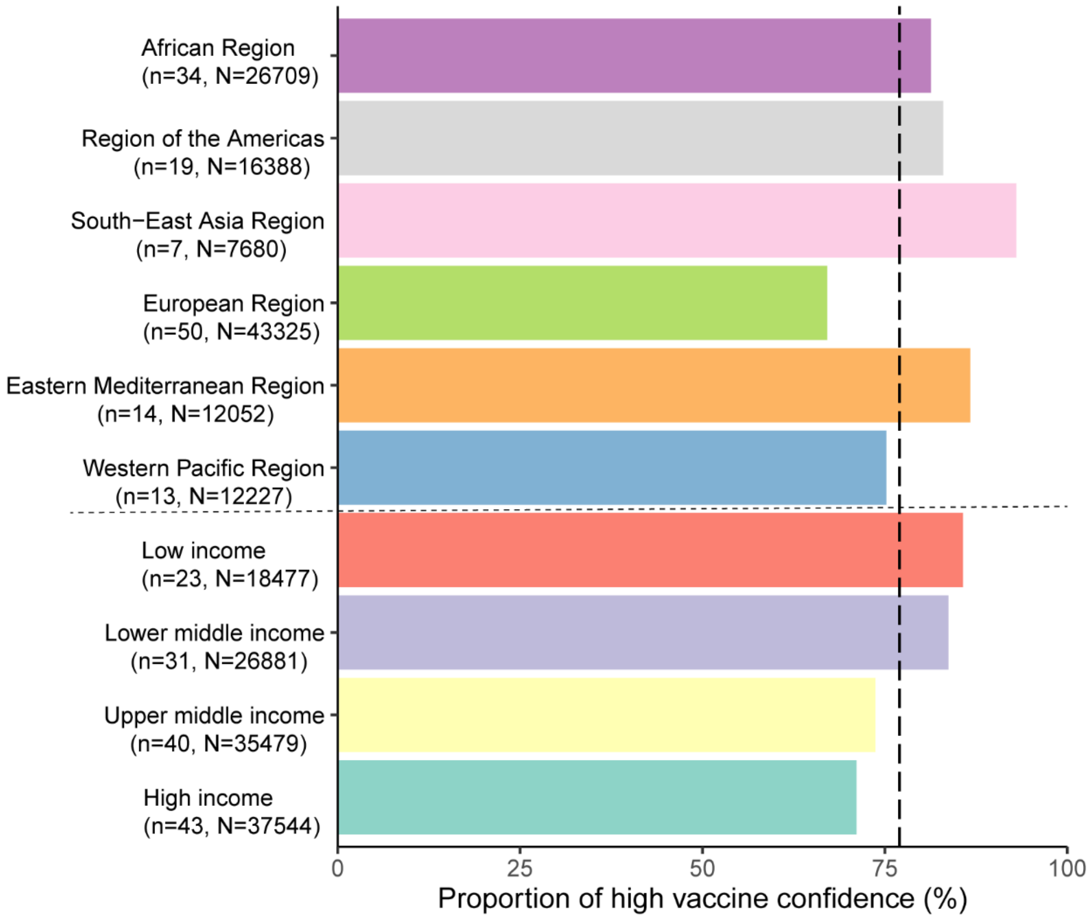

Supplementary Figure 11. Vaccine confidence at the regional level

A total of 3765 individuals from 4 countries were not included in this figure because these four countries are either not WHO member states or are not classified by the World Bank income groups.

Supplementary Table 14. Between-country inequalities in vaccine confidence at the regional level

Region	n	Economic-related concentration index and 95% CI	*p* value	n	Education-related concentration index and 95% CI	*p* value
Country-income group						
Low-income	23	-0.022 (-0.050, 0.007)	0.134	23	0.001 (-0.029, 0.031)	0.943
Lower-middle-income	31	-0.029 (-0.062, 0.003)	0.079	31	-0.034 (-0.066, -0.002)	0.039
Upper-middle-income	40	-0.006 (-0.044, 0.033)	0.768	40	-0.064 (-0.096, -0.032)	<0.001
High-income	43	-0.006 (-0.037, 0.026)	0.724	43	-0.037 (-0.066, -0.008)	0.014
WHO region						
African Region	34	-0.030 (-0.052, -0.008)	0.009	34	-0.017 (-0.040, 0.007)	0.163
Region of the Americas	20	-0.005 (-0.028, 0.018)	0.662	20	-0.008 (-0.031, 0.016)	0.510
South-East Asia Region	7	-0.008 (-0.035, 0.018)	0.504	7	-0.011 (-0.036, 0.015)	0.392
European Region	50	0.010 (-0.025, 0.045)	0.557	50	-0.014 (-0.049, 0.021)	0.431
Eastern Mediterranean Region	14	-0.022 (-0.048, 0.003)	0.089	14	-0.017 (-0.044, 0.010)	0.202
Western Pacific Region	12	-0.049 (-0.128, 0.029)	0.206	12	-0.077 (-0.145, -0.009)	0.034

n represents the number of countries/territories included in the analysis.

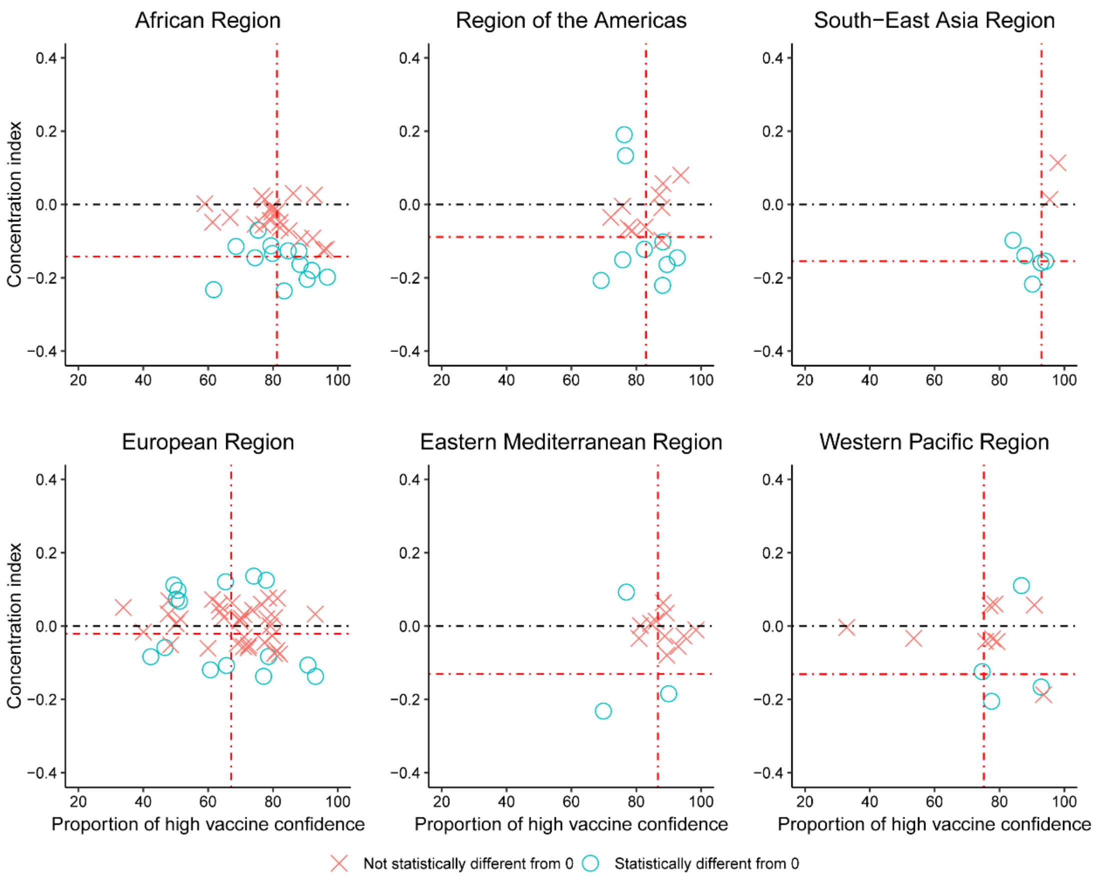

Supplementary Figure 12. Education-related equalities in vaccine confidence in WHO regions

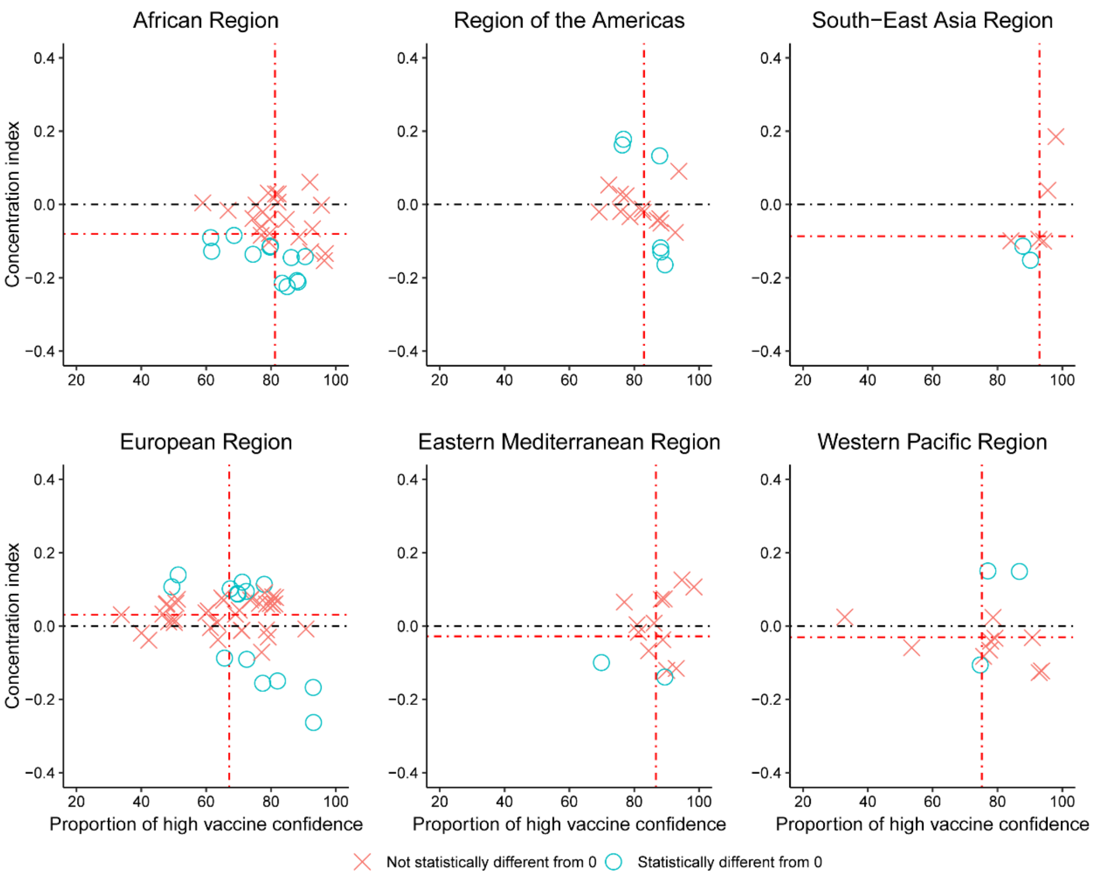

Supplementary Figure 13. Economic-related equalities in vaccine confidence in WHO regions

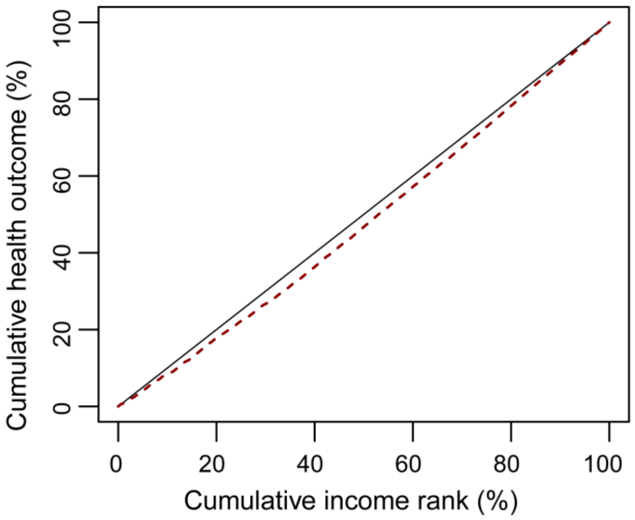

Supplementary Figure 14. A concentration curve based on vaccination coverage and GDP per capita

The y-axis showed the cumulative share of DTP3 vaccination coverage of country in 2015, and the x-axis showed the cumulative share of country ranked by GDP per capita in 2015 from the poorest to the richest. The black 45° line represented a state of perfect equality. The red line represented the concentration curve.

Supplementary Note 1

The equation of calculating concentration index and its variance was followed:

$$\mathrm{C} = \frac{2cov(h_i, R_i)}{\bar{h}} = \frac{1}{n}\sum_{i=1}^{n}\left[\frac{h_i}{\bar{h}}(2R_i - 1)\right] \tag{1}$$

$$\mathrm{Var}(C) = \frac{1}{n^2}\sum_{i=1}^{n}\left(2\frac{h_i}{\bar{h}}\right)^2\left(h_i - \frac{n+1}{2}\right)^2 + \frac{1}{n^2}\sum_{i=1}^{n}\frac{{h_i}^2}{\bar{h}} \tag{2}$$

Where $\bar{h}$ is the mean of the health variable, h_i is the health variable for the i-th individual, R_i is the fractional rank of the i-th individual in the socioeconomic distribution, n is the sample size. We used the standard error and normal approximation to calculate the 95% confidence interval. Variables with a finite upper limit pose challenges in measuring inequality. Bounded variables can be represented either as attainments $a_i \in [a^{min}, a^{max}]$ or as shortfalls from the upper limit $S_i = a^{max} - a_i$. Wagstaff noted that the range of the standard concentration index depends on the mean of the bounded variable and suggested rescaling it to always lie within [−1, 1]. The equation of corrected concentration index was followed:

$$\text{Wagstaff's corrected C} = \frac{1}{n}\sum_{i=1}^{n}\left[\frac{(a^{max} - a^{min})a_i}{(a^{max} - \bar{a})(\bar{a} - a^{min})}(2R_i - 1)\right] \tag{3}$$

Supplementary References

[1] Wagstaff A, Paci P, van Doorslaer E. On the measurement of inequalities in health. Soc Sci Med. 1991;33:545–557.

[2] O'Donnell O, O'Neill S, Van Ourti T, Walsh B. conindex: Estimation of concentration indices. Stata J. 2016;16(1):112-138.

[3] Wagstaff A. The bounds of the concentration index when the variable of interest is binary, with an application to immunization inequality. Health Econ. 2005; 14:429–432.
